# Effects of Composting Different Types of Organic Fertilizer on the Microbial Community Structure and Antibiotic Resistance Genes

**DOI:** 10.3390/microorganisms8020268

**Published:** 2020-02-17

**Authors:** Zeming Zhou, Huaiying Yao

**Affiliations:** 1 Research Center for Environmental Ecology and Engineering, School of Environmental Ecology and Biological Engineering, Wuhan Institute of Technology, Wuhan 430073, China; az1184191208@outlook.com; 2 Zhejiang Key Laboratory of Urban Environmental Processes and Pollution Control, Ningbo Urban Environment Observation and Research Station, Chinese Academy of Sciences, Ningbo 315800, China; 3 Key Laboratory of Urban Environment and Health, Institute of Urban Environment, Chinese Academy of Sciences, Xiamen 361021, China

**Keywords:** antibiotics, organic fertilizer, microbial community structure, ARGs, MGEs, composting

## Abstract

Organic fertilizer is a major carrier that stores and transmits antibiotic resistance genes (ARGs). In the environment, due to the application of organic fertilizers in agriculture, the increasing diversity and abundance of ARGs poses a potential threat to human health and environmental safety. In this paper, the microbial community structure and ARGs in different types of organic fertilizer treated with composting were examined. We found that the abundance and diversity of ARGs in earthworm cast organic fertilizer were the lowest and the highest in chicken manure organic fertilizer. Interestingly, the abundance and diversity of ARGs, especially beta-lactam resistance genes, sulfonamide resistance genes, and macrolide-lincosamide-streptogramin B (MLSB) resistance genes, in organic fertilizers were reduced significantly, while composting caused no significant change in mobile genetic elements (MGEs), where antibiotic deactivation and the use of efflux pumps were the two most dominant mechanisms. It was clear that removal of ARGs became more efficient with increasing reduction in the bacterial abundances and diversity of potential ARG hosts, and integron-mediated horizontal gene transfers (HGTs) played an important role in the proliferation of most ARG types. Therefore, the reduction in ARGs was mainly driven by changes in bacterial community composition caused by composting. Furthermore, rather than HGTs, the diversity and abundance of bacterial communities affected by compost physical and chemical properties were the main drivers shaping and altering the abundance and diversity of ARGs, which was indicated by a correlation analysis of these properties, antibiotic residues, microbial community structure, and ARGs. In general, high-temperature composting effectively removed antibiotic residues and ARGs from these organic fertilizers; however, it cannot prevent the proliferation of MGEs. The insights gained from these results may be of assistance in the safe and rational use of organic fertilizers by indicating the changes in microbial community structure and ARGs in different types of organic fertilizer treated with composting.

## 1. Introduction

Antibiotics have been widely used in disease prevention and treatment for animals and humans. In the mid-1950s, scientists obtained multidrug-resistant bacteria through isolation and culture [1]. At that time, people were aware of the threat of antibiotic-resistant bacteria and drug resistance genes, and they attracted widespread attention. A report published by the WHO states that antibiotic resistance has become increasingly serious and will seriously threaten human health, and it suggests that this topic should receive increasing attention [2]. In current research, ARGs are emerging environmental pollutants and are widely distributed in soil, air, and water, while human activities have promoted the spread of ARGs [3].

Due to the increasing use of antibiotics in agricultural production and livestock breeding, antibiotic resistance has become increasingly severe [4]. Antimicrobials constitute more than 6% of all prescriptions in human medicine, and more than 70% of all consumed pharmaceuticals in veterinary medicine [5]. For example, it is reported that in Turkey, antibacterial drugs used to treat diseased animals and used as a prophylactic or to promote the growth of the undiseased animals account for 33% of the total veterinary pharmaceutical consumption [6]. In Denmark, consumption of antibiotics in 1997 exceeded more than 150 t, out of which >100 t were used as growth promoters [7], while there was an increase of nearly 80-fold in antibiotic usage for growth promotion within a span of four decades in the US [8]. A similar increase in antibiotic usage has been observed in several other countries (e.g., Australia, New Zealand, the EU) [9]. In particular, China uses more than 46% of the world’s antibiotics to ensure growth augmentation and disease control in livestock industries [10,11]. However, only a small concentration of consumed antibiotics can be absorbed because most antibiotics are excreted into the environment through excrement, and manure is considered an important reservoir for environmental antibiotic contamination [12,13]. Therefore, in the environmental microbiota, unprocessed manure contains large amounts of ARGs and antibiotic residues [14,15]. To increase animal immunity and yield, antibiotics (especially tetracyclines (TCs), sulfonamides (SAs), and fluoroquinolones (FLQs)) are widely used as veterinary drugs and growth promoters in the farming industry [16,17]. TCs, SAs, and FLQs have been detected in liquid manure and dung samples at up to 46, 91, and 8.3 mg kg^−1^, respectively [18]. Repeated fertilization of agricultural soils with animal manure can lead to a nonpoint source contamination of the terrestrial environment with these substances, and in turn, accumulation of them in agricultural soils. Studies of the occurrence of various antimicrobials in different soils fertilized with animal manure reported the maximum concentrations of 0.3 mg kg^−1^, 0.015 mg kg^−1^, and 0.37 mg kg^−1^ for TCs, SAs, and FLQs, respectively [18,19,20]. Of all antibiotics, 22% of SAs and 66% of TCs are used in the animal industry. For swine, the frequency of ARGs carried by bacteria seems to be especially high compared to that of other livestock due to the extensive use of antibiotics in these animals. In addition, the concentration of TC reached as high as 300 mg kg^−1^ soil, which demonstrates that repeated fertilization with liquid manure from intensive pig farming leads to the accumulation of this antibiotic [21]. When soil received raw or digested manure, vegetables may be contaminated by antibiotic-resistant bacteria. The misuse and overuse of continuous emissions into the environment of antibiotics has raised concerns over the risk of promoting antibiotic resistance [22,23,24].

In Shandong Province, approximately 30% of the annual agricultural product income comes from the livestock breeding industry, and it trends upward year by year. By the end of 2018, the number of live pigs in livestock farms in Shandong Province was 46.6 million, and studies have found that antibiotics that can be completely absorbed by animals account for less than 70% of the total antibiotics used and that most are excreted [25,26]. In addition, antibiotics affect cell functions, change the expression of virulence factors, and result in the transfer of antibiotic resistance at subinhibitory concentrations. However, resistance to antibiotics is a highly complex process that is not completely understood, even in clinical environments. Therefore, although direct sunlight drying, indoor air drying, composting, and other bioconversion treatments have been used for the utilization and treatment of manure in large livestock and poultry farms [27], livestock manure is widely used in agricultural production and current processes do not have sufficient capacity to address emerging environmental contaminants such as antibiotics, resistant bacteria and ARGs (The 13th Five-Year Plan for the Development of Modern Animal Husbandry in Shandong Province (2016–2020)).

In this work, four livestock manures were studied. Changes in physicochemical properties (pH, C, N, NH_4_^+^-N, and NO_3_^−^-N) and antibiotic residues (sulfadiazine (SDZ), oxytocin (OTC), sulfamethazine (SMZ), chlortetracycline (CTC), sulfamethoxypyridazine (SMN), TC, ofloxacin (Oflox), doxycycline (Dox), and enrofloxacin (Enroflox)) before and after composting were determined. Bacterial 16S rRNA gene high-throughput sequencing was used to investigate changes in bacterial community characteristics; high-throughput qPCR (HT-qPCR) targeting 285 ARGs, 10 mobile genetic elements (MGEs), and 16S rRNA marker genes was used to characterize the ARGs in organic fertilizers before and after compositing. Bipartite network analysis was used to reveal the ARGs shared between uncomposted organic fertilizers and compost-treated organic fertilizers. To explore how antibiotic resistance in organic fertilizers changes before and after composting, the effect of composting on organic fertilizers and the relationships of physicochemical properties, microbial community and ARGs were analyzed. The outcome will provide important theoretical support for the rational utilization of organic fertilizer in agriculture and the effective disposal of wastes.

## 2. Materials and Methods

### 2.1. Manure and Composting

Manure was collected from fertilizer factories in Shandong Province and was cow dung (CD), chicken manure (CM), sheep manure (SM), or earthworm cast (EC) (earthworms were naturally cultured in pure cow dung until the earthworm digestion was completed, and then samples were collected). According to the USEPA (U.S. Environmental Protection Agency) standard for composting, before manures are applied on greenhouse fields as fertilizer, thermophilic (days 1 to 7), mesophilic (days 8 to 25), and maturation (days 26 to 32) stages occur in manure that is piled up and composted for approximately 32 days with the temperature “maintained at 55 °C or higher for 3 d” (USEPA, http:/water.epa.gov/scitech/wasteteclh/biosolids/index.cfim, accessed June 2015), which results in manure that is dark brown, damp, and strong smelling.

When transported to the laboratory, the samples were stored at -20 °C or below for antibiotic concentration determination and genomic DNA extraction. Samples from compost were denoted CDC, CMC, SMC, and ECC. Uncomposted manure samples were denoted CDU, CMU, SMU, and ECU.

### 2.2. Analysis of Physicochemical Properties and Antibiotic Residues

Before analysis of physicochemical properties and antibiotic residues, the samples were freeze-dried (Labconco, Kansas City, MO) and homogenized by sieving through a 0.2 mm mesh.

Five grams of sample (dry weight) was mixed well with 12.5 mL UltraPure water (a soil-to-water ratio of 1:2.5) and subjected to pH measurement (pH meter, Delta 320, Mettler Toledo, USA). NH_4_^+^-N and NO_3_^−^-N in the samples were extracted with 2 M KCl and measured by a continuous flow analyzer (SAN plus, Skalar Analytical B.V., The Netherlands). Approximately 100 mg of organic fertilizer samples was used to determine their C, H, O, N, and S contents and C/N ratios by means of an elemental analyzer (vario MACRO cube, Elementar, Germany) [28].

The antibiotic residues were determined by high-performance liquid chromatography-tandem mass spectrometry (HPLC-MS/MS, Thermo Fisher Scientific, Waltham, USA) analysis, and extraction and purification procedures followed the description by Qian et al. [29,30] Nine different antibiotics were analyzed: SDZ, SMZ, SMN, OTC, TC, CTC, Dox, Oflox, and Enroflox. In this experiment, according to the parameters of the test instrument, the detection limit was in the range of 0.5–15 μg kg^−1^ manure (dry weight), and the limit of quantification was in the range of 1.5–50 μg kg^−1^ manure (dry weight) [29].

### 2.3. DNA Extraction from Organic Fertilizer

Two hundred milligrams of each sample was used for DNA extraction by using a Fast DNA SPIN Kit for Soil (MP Biomedicals, USA), and the procedures were performed according to the manufacturer’s instructions. To remove humic acid, 5.5 M guanidinium isothiocyanate (Amesco) was added before the DNA solution was transferred to the SPINTM Filter Tube. Then, 50 μL of DES (diethyl sulfate) solution from the kit was used to elute the fertilizer DNA. The concentration and quality of extracted DNA were checked using 1.0% agarose gel electrophoresis and spectrophotometric analysis (Nanodrop ND-1000, Thermo Fisher Scientific, Waltham, USA). Finally, the extracted DNA was preserved at −20 °C for further analysis.

### 2.4. 16S rRNA Gene Amplification, Illumina Sequencing, and Bioinformatic Analysis

The organic fertilizer DNA samples were amplified using the universal primers 314F (5′- CCTAYGGGRBGCASCAG-3′) and 806R (5′- GGACTACNNGGGTATCTAAT-3′) to target the hypervariable V4−V5 region of the bacterial 16S rRNA gene in a 20 μL reaction system (Gene Amp^®^ 9700, ABI). Each 25 μL PCR contained 12.5 μL of 2 × T5 super PCR Mix, 1 μL of forward primer (10 μM), 1 μL of reverse primer (10 μM), 10 ng of template DNA, and nuclease-free water to a volume of 25 μL. The conditions of PCR amplification and recovery of purified products were performed as follows: first, the sample was incubated at 98 °C for 3 min to activate the PCR enzyme. Then, 35 cycles of 98 °C for 10 s, 55 °C for 10 s, and 72 °C for 30 s were performed. The PCR product was then extended at 72 °C for 2 min and maintained at 10 °C until the incubation was halted by the user. The Universal DNA Purification kit (Tiangen, Beijing, China) was used for the purification of PCR products, and a NanoDrop spectrophotometer (ND-1000, Thermo Fisher Scientific, Waltham, MA, USA) was used to quantify the PCR products. Premixed samples were sent for sequencing at Origingene (Shanghai Origingene Bio-pharm Technology Co., Ltd., China) on an Illumina PE250 platform. Raw paired-end reads were assembled, and clean joined reads were first generated by the Beijing Genetics Institute (BGI) [31,32]. Then, Quantitative Insights Into Microbial Ecology (QIIME) was used to process and analyze the generated high-quality sequences [33], and the open-reference operational taxonomic unit (OTU) was defined at the 97% similarity level by UCLUST clustering [34]. To obtain the taxonomy of representative sequences, this study uses an RDP (Ribosomal Database Project) classifier based on the Silva v.119 16S rRNA gene database (http://www.arb-silva.de).

### 2.5. Real-Time Quantitative PCR and High-Throughput Quantitative PCR Analysis

To obtain total bacterial abundances, this study used real-time quantitative PCR (RT-qPCR) (Light Cycler 480 II) (Roche Scientific, Indianapolis, IN, USA) with the universal primers 515F (5′-GTGCCAGCMGCCGCGG-3′) and 907R (5′-CCGTCAATTCMTTTRAGTTT-3′) for amplification of the bacterial 16S rRNA gene in triplicate. All assays were conducted in a 20 μL qPCR system consisting of 10 μL of 2 × TransStart Top Green qPCR SuperMix (AQ131, Transgen Biotech, Beijing, China), 0.5 μL of each primer (10 μM concentration, 0.5 μM final), 2 μL of DNA as a template, and 7 μL of HyPure Molecular Biology grade water (Thermo Fisher Scientific, Waltham, USA). The thermal profile of RT-qPCR was as follows: the initial enzyme activation was performed at 95 °C for 5 min, and then 40 cycles of the following procedure were used for amplification: 95 °C for 15 s, 60 °C for 60 s, and 72 °C for 20 s. A negative control group used three RNase-free water samples as the template in reactions. To obtain a standard curve, *Escherichia coli* was used to clone the 16S rRNA-encoding gene, which was transferred to a plasmid as a target gene, and then 10-fold gradient dilution was performed (amplification efficiency 96−104%, r^2^ > 0.99). In this experiment, the 10-fold gradient dilution of the plasmid standard solution was processed together with the sample and tested. The number of 16S rRNA-encoding genes in the sample was quantified by a standard curve generated by the plasmid.

The relative abundance of ARGs was determined by using real-time PCR (Wafergen SmartChip Real-time PCR system) (Wafergen, Fremont, CA). The primers used in this experiment targeted almost all major categories of ARGs and MGEs (8 transposases, class 1 integrons, clinical class 1 integrons) and the 16S rRNA genes, for a total of 296 targets (Table S2) [35,36]. The qPCRs were performed as previously described: 100 nL (50 nL from an assay source plate and 50 nL from a sample source plate) containing the required 1x Light Cycler 480 SYBR Green I Master (Roche Scientific, Indianapolis, IN, USA), RNase-free PCR-grade water, each primer and DNA template was added per well. The wells need to be heated to 95 °C for 10 min to activate the enzyme, and then 40 cycles were performed according to the following procedure: 30 s at 95 °C and 30 s at 60 °C [35]. The qPCR results were obtained directly by Wafergen software. Analyzing the experimental results identified samples with multiple melting peaks and amplification efficiencies beyond the range of 1.8–2.2, which were then not used. The experiment used a threshold cycle (Ct) of 31 as the detection limit, and ARGs amplified in all replicates could be used. The relative copy number was calculated according to Equation (1), and the absolute copy number was calculated from the 16S rRNA gene copy number according to Equation (2) [37,38,39,40,41].

(1) $$\mathrm{Relative}\;\mathrm{Gene}\;\mathrm{Copy}\;\mathrm{Number}=10^{\frac{31-C_{T}}{10/3}}$$

(2) $$\mathrm{Normalized}\;\mathrm{gene}\;\mathrm{Copy}\;\mathrm{Number}=\frac{\mathrm{Relative}\;\mathrm{ARG}\;\mathrm{Gene}\;\mathrm{Copy}\;\mathrm{Number}}{\mathrm{Relative}\;16s\;\mathrm{rRNA}\;\mathrm{Gene}\;\mathrm{copy}\;\mathrm{number}}\times 4.1$$

where 4.1 was considered the average number of 16S rRNA gene copies per bacterium based on the Ribosomal RNA Operon Copy Number Database.

### 2.6. Statistical Analysis

Data analysis was performed for triplicate samples. and the mean values with standard errors are presented in the figures. The raw data for the microorganisms (diversity, composition, etc.) were analyzed by using R 3.3.Network analysis was performed with Python 3.7 using the interactive platform Gephi with the Fruchterman Reingold placement algorithm [42,43]. Microsoft Excel was used for the calculation and collation of data. IBM SPSS Statistics was used for statistical tests, and differences were considered significant at *p* < 0.Quantitative data is expressed as mean ±SD and analyzed by one-way ANOVA. The post-hoc LSD (Least Significant Difference) test was used to compare differences between groups. The bar charts, scatter diagrams, pie charts, and heatmaps were generated by OriginPro. Redundancy analysis was performed using CANOCO 5.

## 3. Results

### 3.1. Antibiotic Residues in Organic Fertilizer

Because of sorption, antibiotics are often concentrated in the solid phase of manure [44,45,46,47]. In addition, the half-lives of different antibiotics in the manure varied [48,49], and the anticipated storage period of manure was longer than these half-lives. This result indicated that before manure was used for agriculture, the parent antibiotic compounds may have degraded.

Figure S1 shows the degradation of antibiotics in the four groups of organic fertilizers before and after composting. With the completion of composting, TCs, SAs, and quinolones in the CM group were obviously degraded. In the CM group, the removal rates of OTC, Dox, Oflox, and SMN reached more than 60%. At the same time, the OTC concentration in the CM samples decreased significantly, and the final elimination rate reached 100%. It has been reported that during manure composting, OTC may degrade with a total removal of over 90%, which is consistent with our results. The antibiotic residues in the EC group and SM group were lower than the detection limit, so they were not detected. The Oflox (a quinolone) removal rate in the CM group organic fertilizer was 48%. The Enroflox removal rate was 26.7%. TC, CTC, and SDZ were not detected, probably due to the low levels remaining.

### 3.2. Physicochemical Properties of Organic Fertilizer

Composting can significantly reduce the pH of organic fertilizers compared to that of uncomposted fertilizers (Figure S2). However, in the CM organic fertilizer, the pH increased after composting. The initial value was 7.36, and the pH rose to 7.59 after composting. The organic fertilizers that were not composted had a pH of 7 or higher. After composting, the pH of the organic fertilizers fell below 7.

When composting, the organic matter in the manure is generally utilized and converted by microbes by humification or mineralization. In addition, the microbial activity can be reflected by variation in the C/N ratio; obviously, the C/N ratio of the ECC, CDC, and SMC groups were lower than that of groups without composting (Figure S3). Conversely, the C/N ratio in the CM group after composting was significantly higher than that in the uncomposted samples.

### 3.3. Structure and Characteristics of Microorganisms in Organic Fertilizer

#### 3.3.1. Diversity of Microorganisms

**Figure 1:**
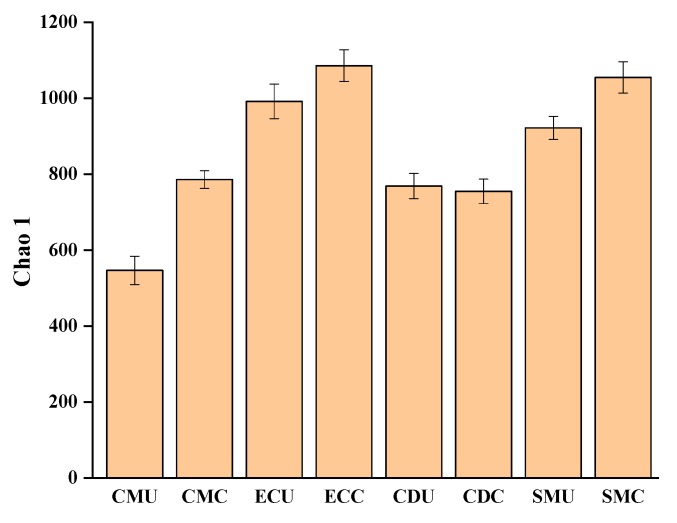
Richness of bacterial communities in organic fertilizers using the Chao1 estimator. Samples from compost were denoted CDC, CMC, SMC, and ECC. Uncomposted manure samples were denoted CDU, CMU, SMU, and ECU.

After assembly and quality filtering, 389,667 high-quality sequences were found in all samples, and a total of 1913 OTUs were revealed by statistical analysis of the biological information. A community abundance (Chao1) analysis demonstrated that the composting treatment significantly increased microbial abundance relative to that of uncomposted samples. The abundance of organic fertilizers increased after composting, especially in the CM, EC, and SM groups (Figure 1).

#### 3.3.2. Composition of Microorganisms

Venn diagrams demonstrate unique OTU distribution with significant differences between the uncomposted and composted groups. Figure S4a shows that there are 251 common OTUs in the four compost-treated samples, while in the uncomposted treatment group there are only 59 OTUs shared by the four groups. Figure S4a,b together showed that before the composting treatment, the OTUs with significant differences among the four samples accounted for the vast majority, reaching 441 OTUs in the ECU group, but after the composting treatment the common OTUs in the four samples increased significantly.

**Figure 2:**
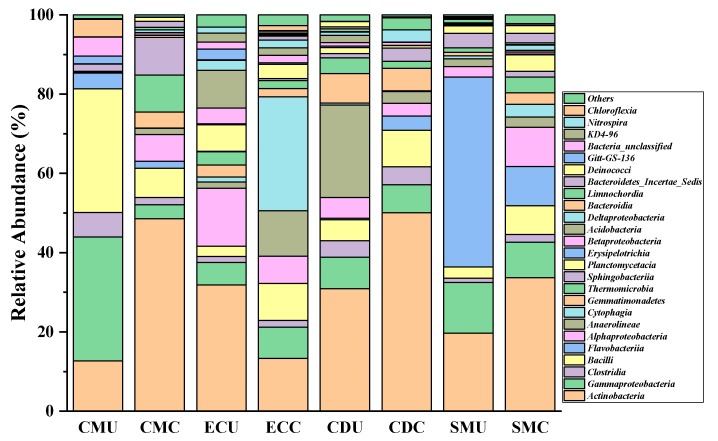
The effect of composting on the composition of organic fertilizer microbial community. The composition of the microbial community at the class level. Only the most abundant taxa (>1% genus) are displayed. The order of the genes is based on their relative abundance (mean, *n* = 3).

In the CM group, *Proteobacteria* (36.7%) and *Firmicutes* (39.4%) were the main phyla in the uncomposted samples, but after composting *Actinobacteria* was the dominant phylum in the CM, CD, and SM groups. Clearly, composting was conducive to increasing the relative abundance of *Actinobacteria* while decreasing its relative abundance in the EC groups (Figure S5 and Figure 2).

#### 3.3.3. Correlation between Physicochemical Properties and Microorganisms

**Figure 3:**
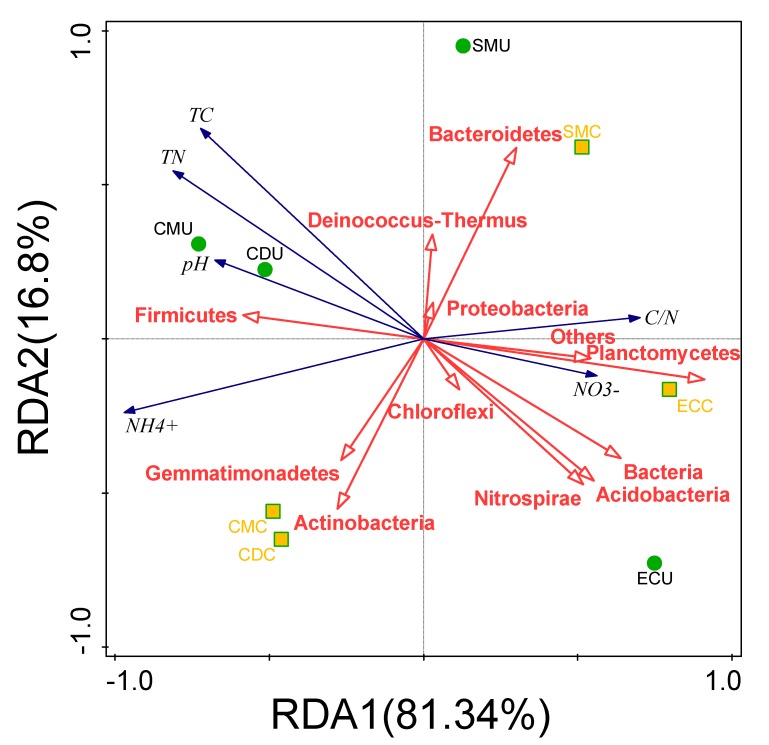
Redundancy analysis (RDA) of the correlations between physicochemical properties of organic fertilizer samples before and after composting and major microbial phyla (>1%) (*Actinobacteria, Firmicutes, Proteobacteria, Bacteroidetes, Chloroflexi, Gemmatimonadetes, Planctomycetes, Acidobacteria, Deinococcus-Thermus, Bacteria, Nitrospirae*). TN: total nitrogen; TC: total organic carbon.

As redundancy analysis shows (Figure 3), *Planctomycetes*, *Bacteria*, *Acidobacteria*, and *Nitrospirae* relative abundances were positively correlated with NO_3_^−^ (*p* < 0.01) but negatively correlated with total organic carbon (TC) and total nitrogen (TN). In contrast, *Firmicutes* were significantly positively correlated with NH_4_^+^ and negatively correlated with NO_3_^−^. Among the fertilizer groups, CM and CD showed obvious clustering before and after composting.

### 3.4. ARGs in Organic Fertilizer

#### 3.4.1. Diversity of ARGs and MGEs in Organic Fertilizer

**Figure 4:**
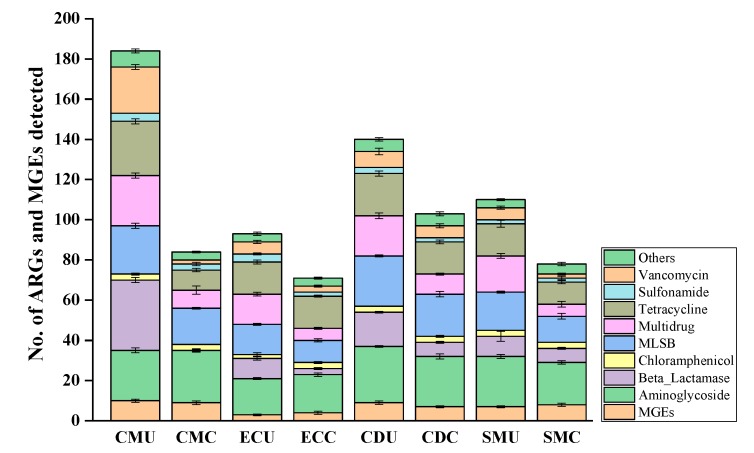
The detected number of antibiotic resistance genes (ARGs) and mobile genetic elements (MGEs) in the four sets of organic fertilizer samples, includes CMU and CMC, ECU and ECC, CDU and CDC, SMU and SMC. MLSB: macrolide-lincosamide-streptogramin B.

The organic fertilizer samples exhibited a total of 228 ARGs (Figure 4), and up to 184 and 71 ARGs were detected in CMU and ECC, respectively. Although composting resulted in an average of 96 ARGs in the four composted samples, up to 103 ARGs were detected in the CDC sample. This result indicated that ARGs existed widely in organic fertilizer and that the incidence of ARGs was different in different organic fertilizer samples. Manure application not only adds nutrients and organic matter to cultivated soil for crop growth but also introduces ARGs, posing a potential risk to human health.

**Figure 5:**
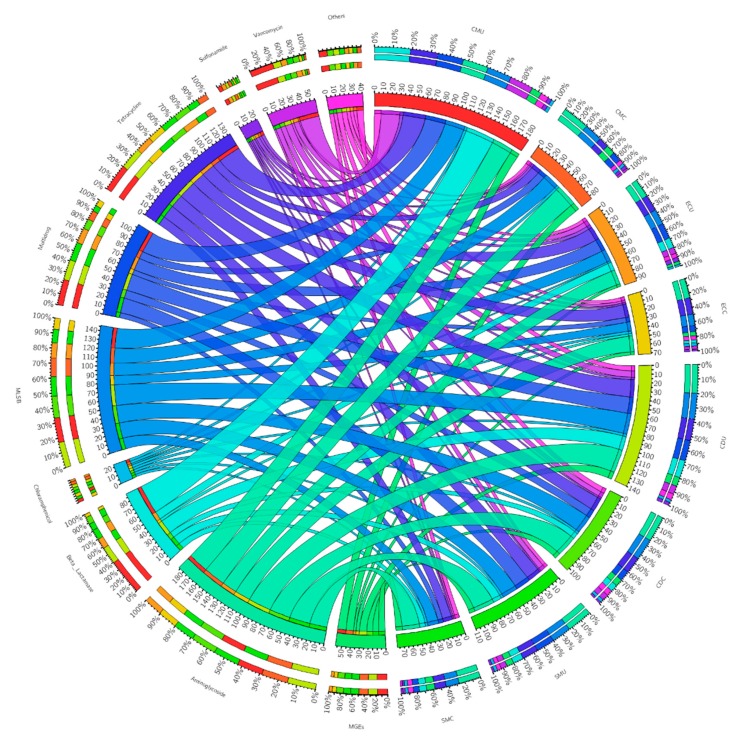
Distribution of each ARG type in eight organic fertilizer samples. The data were visualized via Circos software (http://circos.ca/). The length of the bars of each sample on the outer ring represents the percentage of ARGs in each sample.

As shown in Figure S6, antibiotic deactivation accounted for 41.74% of resistance mechanisms, efflux pumps accounted for 29.36%, and cellular protection resistance mechanisms accounted for 27.06% in the uncomposted samples. The detected ARGs could potentially confer resistance to all the major antibiotics. Of the targets of all the detected ARGs, aminoglycoside, beta-lactam, multidrug, macrolide-lincosamide-streptogramin B (MLSB), TC, and vancomycin antibiotics are important for human medicine (Figure 5), and even resistance genes for the “last-resort” life-saving antibiotic vancomycin were detected [50]. The incidence of ten types of ARGs was determined in organic fertilizer samples (Figure 5). Resistance mechanisms targeting aminoglycosides (7.5–11.6%), TC (5.2–15.6%), multiple drugs (4–16.7%), beta-lactam ((not detected)~28%), and MLSB (5.7–12.9%) were the five most common types in organic fertilizer samples, followed by resistance mechanisms for vancomycin (2.9–32.9%), SA (6.9–13.8%), chloramphenicol (6.9–10.3%), and MGEs (5.2–13%). The incidence of ARGs differed with organic fertilizer sample.

#### 3.4.2. Abundance and Enrichment of ARGs and MGEs in Organic Fertilizer

**Figure 6:**
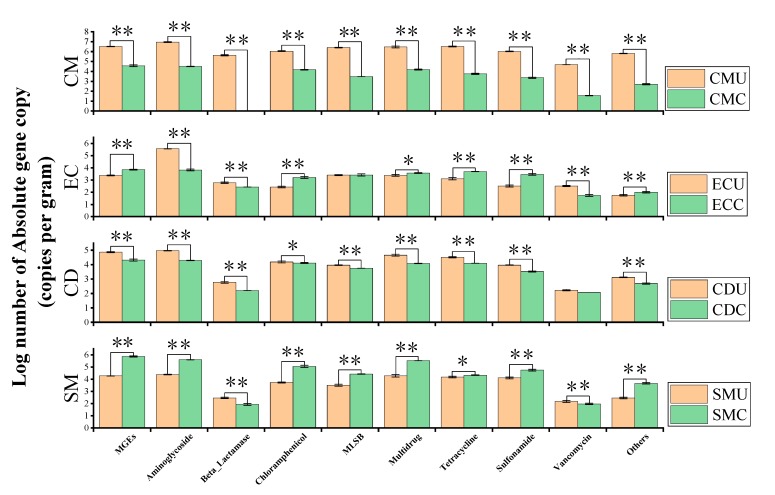
Log number of absolute gene copy number (copies per gram) of ARGs and MGEs. The histogram showing the distribution of different types of ARGs (classified by the classes of antibiotics that they resisted) and MGEs in the four groups of organic fertilizers before and after composting. ** (*p* < 0.01) on the bar indicates a statistically significant difference. * (*p* < 0.05) on the bar indicates a statistically significant difference.

The absolute abundance of ARGs in organic fertilizer samples ranged from 2.3 × 10^4^ to 2.49 × 10^7^ copies g^−1^ solid dry weight (Figure 6). Figure 6 shows that after composting treatment, the content of ARGs in CM and CD groups is reduced, but the EC and SM samples show opposite trends. In EC samples and SM samples, MGEs increased after composting, which was the same trend as that found for ARGs.

Because of the different numbers of bacterial cells in organic fertilizer samples, the normalized copy numbers of ARGs and MGEs were calculated relative to the 16S rRNA gene copy number (Figure S7). Obviously, composting reduced ARGs in organic fertilizers, but ARGs in SM samples increased after composting, as did MGEs. In addition, we observed that EC samples had less ARG richness and diversity than other samples, and their abundance of MGEs was much lower than that of other samples.

**Figure 7:**
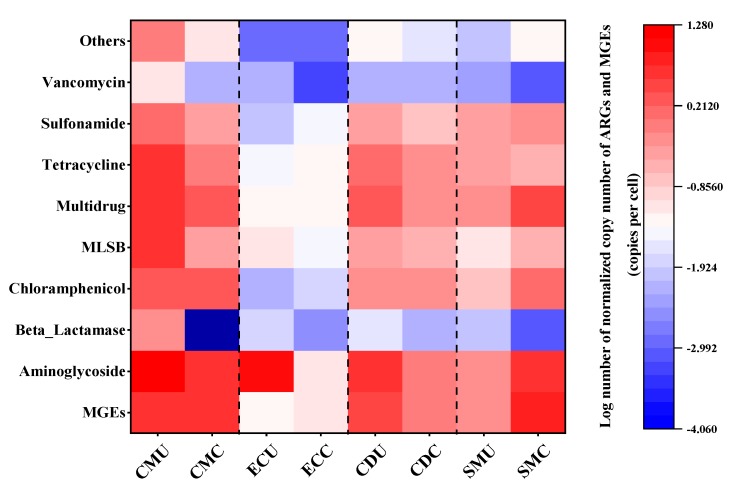
Resistance gene profile from eight organic fertilizer samples. Each column is labeled with a sample name, and each row is the result from a single primer set. Resistance profiles that confer resistance to all major classes of antibiotics included resistance to aminoglycoside, beta-lactamase, chloramphenicol, MLSB, multidrug, tetracycline, vancomycin, and sulfonamide.

According to a heat map analysis (Figure 7), the total abundance of ARGs in the samples after organic fertilizer composting was lower than that before organic fertilizer composting. However, in the SM group of organic fertilizers, the abundance of multidrug, MLSB, chloramphenicol, sulfonamide, aminoglycoside, ARGs, and MGEs after composting was higher than that of uncomposted fertilizers, and the same phenomenon appeared in the EC group for chloramphenicol, sulfonamide, ARGs, and MGEs.

In view of the different amounts of antibiotics added to feed used in poultry farming, the abundance of native ARGs in the intestines of poultry also varies. Although the abundance of some ARGs after composting is higher than that before composting in this study, the overall results show that the abundance of most ARGs after composting is significantly reduced. ARGs from different sources of organic fertilizers are not exactly the same, but overall, aminoglycoside resistance genes and MGEs are relatively abundant.

**Figure 8:**
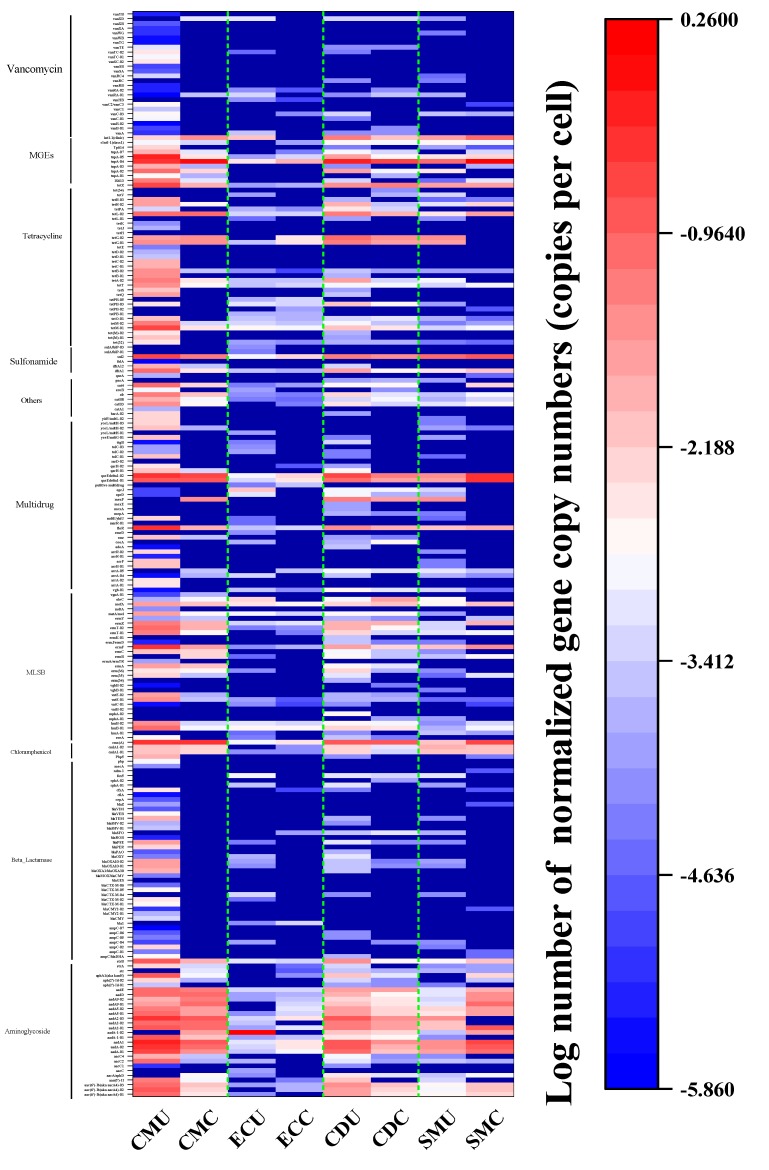
Heatmap analysis of ARGs in organic fertilizer samples. The vertical axis lists the detected ARGs found in this study. The order of the genes was based on their similarity abundance.

Figure 8 shows the enrichment in ARGs in each organic fertilizer sample, and the total enrichment in organic fertilizer samples was approximated for all ARGs. The absolute gene copy number of ARGs ranged from 0 to 313,328.57 (the CMU sample). This result showed that the antibiotic resistance in different manures showed large discrepancies and that the variation in different antibiotic types varied clearly among organic fertilizer samples, especially those without composting which had the highest abundance.

The detailed change in each subtype of ARGs is shown in Table S3. The aminoglycoside resistance genes and MGEs had the most significant changes in total abundance, and the total abundance in the uncomposted samples was significantly higher than that in the composted samples. For MGEs, *tnpA-04* was the most abundant gene in all samples. For TC resistance genes, the total abundance in the CMU sample was the highest, with *tetX*, *tetG-02*, and *tetG-01* being the three most abundant genes compared with their abundance in the uncomposted samples. For multidrug resistance genes, *floR*, *qacEdelta1-01*, and *qacEdelta1-02* had higher abundances in CMU and CDU samples than in CMC and CDC samples. For MLSB resistance genes, CMU samples had a high abundance and the abundance in uncomposted samples was significantly higher than that in composted samples. For chloramphenicol resistance genes, *cmx(A)* was the most abundant gene in all samples.

#### 3.4.3. Correlation between ARGs and Microorganisms

**Figure 9:**
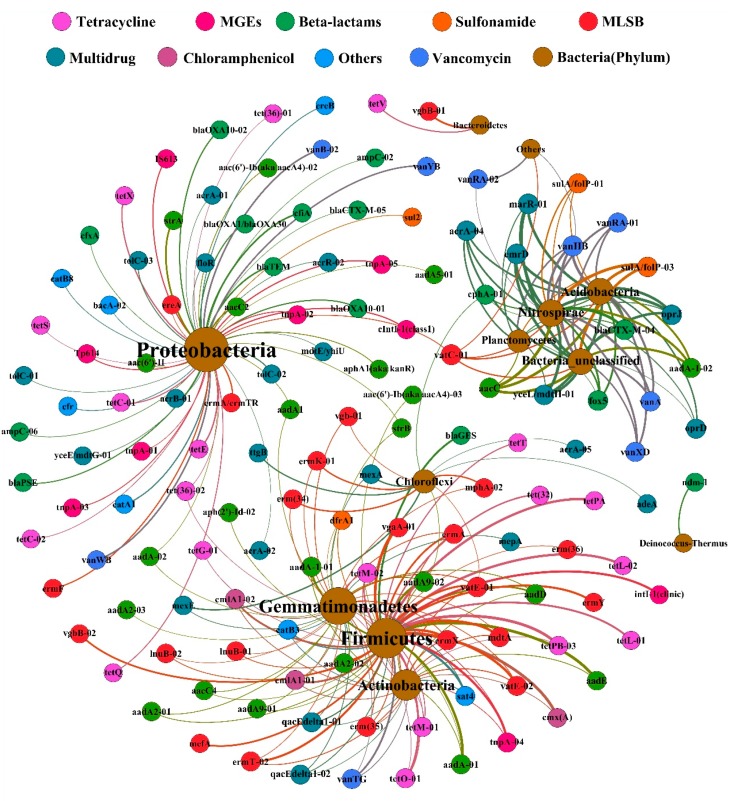
Network analysis of cooccurrence between ARGs, MGEs, and bacteria. Relationships between ARGs, MGEs (relative gene copy number), and bacteria (at the phylum level, 16S rRNA gene sequence data) based on Pearson’s correlation coefficients (*p* < 0.05). The nodes are colored according to ARG class and phylum, and the node size is dependent on the number of connections to other nodes (degree). Each connection represents a significant correlation (*p* < 0.05), and the edge line width represents the corresponding Spearman’s correlation coefficient.

The cooccurring ARGs, MGEs (relative gene copy number) and potential host bacteria (at the phylum level, 16S rRNA gene sequence data) based on Pearson’s correlation coefficients (*p* < 0.05) were analyzed by network analysis (Figure 9). As Figure 9 shows, bacteria in the phyla *Actinobacteria* and *Firmicutes* showed close relationships with several ARG types (such as TC, MLSB, and intl-1 (clinic) ARGs). In addition, SA resistance genes, TC resistance genes and integrase genes showed significant and positive correlations with *Proteobacteria*; six multidrug resistance genes (*marR-01*, *yceL/mdtH-01*, *emrD*, *acrA-04*, *oprJ*, and *oprD*) and five vancomycin resistance genes (*vanXD*, *vanHB*, *vanRA-01*, *vanA*, and *vanRA-02*) showed significant and positive correlations with *Nitrospirae*; however, the relationship between *Bacteroidetes* and the ARGs was generally weak. When there was a strong and significant positive correlation between ARGs and coexisting microbial populations, it can be speculated that the cooccurrence pattern between ARGs and microbial populations is nonrandom, further indicating possible host information for ARGs. Thus, *Actinobacteria*, *Firmicutes*, *Proteobacteria*, and *Nitrospirae* were identified as potential hosts for ARGs.

**Figure 10:**
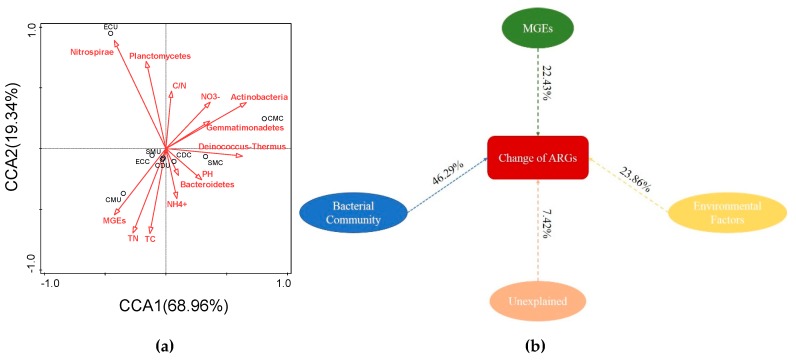
(**a**) Canonical correspondence analysis (CCA) illustrating relationships between microbial phyla, ARGs and environmental factors, including total nitrogen, total carbon, and pH. The percentage of variation explained by each axis is shown, and the relationship is significant (*p* < 0.01) based on 999 permutations. (**b**) Variation partitioning analysis (VPA) differentiates the effects of bacterial communities, environmental factors, and mobile genetic elements (MGEs) on ARG profile alterations. TN: total nitrogen; TC: total organic carbon; and MGEs: mobile genetic elements.

The relationships between environmental factors, MGEs, microbial communities, and ARGs were explored by performing canonical correspondence analysis (CCA) (Figure 10). The microbial communities at the phylum level and MGEs were analyzed as the environmental factors for ARGs, and the first two principal components (PCs) accounted for 88.3% of the total variation. The pH and NO_3_^−^ exhibited a significant positive relationship with the abundance of ARGs in fertilizer. Additionally, TN, TC, and the C/N exhibited no significant correlation with ARGs. Four phyla (*Deinococcus-Thermus, Actinobacteria,* *Gemmatimonadetes, and Bacteroidetes*) were found significantly correlated with the ARGs in fertilizer (*p* < 0.05). *Deinococcus-Thermus*, *Actinobacteria*, and pH positively correlated with the first axis (explaining 68.96% of total variance) and SMC and CMC samples. However, the phyla *Nitrospirae* and *Planctomycetes* were negatively correlated with the first axis but positively correlated with the second axis (explaining 19.34% of total variance) and the ECU sample. Variation partitioning analysis (VPA) was used to determine the key contributor of bacterial communities and MGEs to the variation as whole or separate factors (Figure 10), and the variation was 92.58%, which could explain the selected variables; the bacterial community contributed 46.29% of the total ARG variation, which was higher than the contributions of environmental factors (23.86%) and MGEs (22.43%). The Mantel test (Bray−Curtis distance, r = 0.4959, *p* < 0.05) showed that, regardless of composting, a significant correlation between bacterial communities (OTUs) and ARG profiles was obvious.

## 4. Discussion and Conclusions

Through an analysis of physicochemical properties and antibiotic residues and comparing different treatment methods and organic fertilizers, it can be seen that composting can significantly reduce the antibiotic residues in organic fertilizers. The degradation of SMN during the composting of animal manure is inconsistent. This result was in good agreement with the results of the last two studies: dairy cow manure can effectively decompose the residual SMN after composting, which is speculated to be caused by the combined effects of raw material solids and temperature [51]. At the same time, the results of He’s research showed that the SA and SDZ remaining in the broiler manure were almost completely degraded after composting. This phenomenon may be caused by temperature-dependent abiotic processes [52]. Composting can effectively degrade residual antibiotics in organic fertilizers but not completely remove them. The possible reason was that the antibiotic residue decayed significantly in the manure after the treatment, resulting in decreased antibiotic residue [29]. Previous studies showed that the target antibiotics were removed, while the proliferation of ARGs could not be prevented, and there is a significant positive correlation between the residues of antibiotics at subtherapeutic levels and the accumulation of ARGs [53], and that their degradation also has a positive effect on maintaining the presence of ARGs [54]. The addition of antibiotics inhibits microbial activity in the early stages of composting but promotes the proliferation of ARGs, especially in the mesophilic phase. Integron-mediated horizontal gene transfer (HGT) plays an important role in the proliferation of most ARG types studied [55,56,57,58,59]. Therefore, as antibiotic residues still exist in organic fertilizers, their effects on ARG proliferation cannot be ignored.

After composting, the C/N changes of EC, CD, SM, and CM groups indirectly reflected their microbial activity. Analyzing the differences in microbial community structure of organic fertilizer indicated that there are obvious differences in the microbial community structures of different organic fertilizers and that composting obviously changes the microbial community structure in organic manure. The manure-borne bacterial community showed that the relative abundance of *Actinobacteria* increased in the EC (13%), CD (50%), SM (33.7%), and CM (48.5%) groups after composting (Figure S5).

To evaluate the effect of compost on ARGs, we used HT-qPCR to quantify the abundance and diversity of ARGs. Although the types and abundances of the 285 assessed ARGs differed greatly from those found in organic fertilizer, a total of 218 ARGs and 10 MGEs were detected, which was consistent with previous studies [11,60] and indicated the importance of animal manure as a reservoir for ARGs [61,62,63]. ARGs for broad-spectrum antibiotics were detected, and some of these ARGs have never been previously reported in organic fertilizer samples [64,65,66,67]. However, this broad-spectrum distribution of ARGs in organic fertilizer samples varies from that in soil samples [55,68]. Although the relative abundance varied among different organic fertilizer samples, the types of ARGs were roughly the same. This is attributable mainly to the different antibiotic contact histories of the two environments [69,70]. Furthermore, previous studies have found that thermophilic anaerobic digestion decreased ARGs better than moderate and mesophilic anaerobic digestion [71,72]. Illumina sequencing of the bacterial 16S rRNA gene in this study showed that after organic fertilizer composting, the diversity and abundance of the microbial community was improved, the dominant population changed significantly (specifically, in the CMC group, *Actinobacteria* (48.5%) became the dominant population, in the CDC group, *Actinobacteria* (50%) is the dominant population and in the SMC group, *Actinobacteria* (33.7%) is the dominant population, while in the ECC group, *Cytophagia* (28.8%) and *Actinobacteria* (13.3%) became the dominant populations). Combining these experimental results, it can be found that after organic fertilizer composting, the diversity and abundance of ARGs was significantly reduced (ranged from 2.3 × 10^4^ to 2.49 × 10^7^) and there was a significant correlation between ARG structure and bacterial community composition (*p* < 0.05) (Figure 9).

The experimental results showed that no complete elimination of ARGs occurred with and without composting. According to the experimental conditions before and after composting, it can be speculated that the dynamic change in ARGs may be attributed to the HGT produced by bacterial cells through MGEs, the changes in the concentrations of residual antibiotics, the changes in NH_4_^+^-N and NO_3_^−^-N content, the temperature change during compost, and the succession of the related bacterial communities. Previous studies have found that reducing MGEs plays a key role in the removal of ARG from compost, which is consistent with our results [73]. In addition, the integrase gene copy number remained nearly constant after composting, which may reflect no significant change in the integron-mediated HGT activity with composting. Current research has found that these relatively abundant ARGs are often detected in organic fertilizers [65]. Clearly, the enrichment in ARGs without composting was much higher than that with composting, which may be due to the many antibiotic residues and ARGs in poultry manure, as poultry manures are regarded as reservoirs of ARGs [39,55,65]. Of course, the amount of antibiotics added to the feed used in different poultry farms is different, and the abundance of native ARGs in the intestines of poultry is different, which may lead to this phenomenon. Although the abundance of some ARGs increased after composting, the overall results showed that the abundance of most ARGs after composting was significantly reduced. Organic fertilizers from different sources have unique ARGs, but overall, aminoglycoside resistance genes and MGEs exhibited high abundance. Existing research results show that composting can effectively reduce most types of ARGs in livestock manure [65]. According to the composting conditions, the temperature increase in the thermophilic phase during the composting process and the exposure to the air during the composting process are not conducive to the survival of anaerobic bacteria, which may lead to a reduction in ARGs after organic fertilizer composting [74]. Due to the anaerobic environment in the intestines of animals and the existence of a large number of Gram-negative bacteria that can carry various ARGs, these ARGs generally do not exist in thermophiles; therefore, composting organic manure for poultry and livestock can effectively reduce [65] ARG content.

According to the Pearson’s correlation coefficients in this study (*p* < 0.05), the correlation between ARGs (qPCR data) and bacterial community structure and composition (16S rRNA OTU data) was significant (Figure 3, Figure 4, and Figure 9). After composting, ARGs showed a significant correlation with the relative abundance of *Firmicutes* and *Actinobacteria*, and the relative abundance of *Actinobacteria* clearly increased (Figure S5 and Figure 9). Previous studies have found that *Bacilli* and *Flavobacteria* within *Firmicutes*, which prevail during the thermophilic phase of composting, were significantly related to ARGs [75]. Many previous reports have shown that [76] *Actinobacteria* exhibit multiresistance and self-resistance [77] and that these bacteria are regarded as the main host microbial community of ARGs. *Firmicutes* and *Actinobacteria* are some of the most prevalent predicted source phyla of ARGs and have a clear correlation with changes in the abundance and diversity of ARGs based on the main resistance mechanisms of acetyltransferases and phosphotransferases [78]. This may also cause aminoglycoside resistance genes to be enriched in organic fertilizers after composting.

These results suggested that rather than HGT, the diversity and abundance of bacterial communities affected by physical and chemical properties were the main drivers of shaping and altering the abundance and diversity of ARGs. Previous studies have shown that bacterial community composition is the main determinant of soil ARG content, which is consistent with the results of this study [78]. As the VPA shows, the percentage of variation explained by MGEs is only 22.43%, which is lower than the percentage explained by the bacterial community and environmental factors. This result indicated that the HGT of ARGs before and after composting is less important than other factors in this study. However, the microbial community structure before and after the composting of different organic fertilizers is very different, and the diversity and abundance of most samples after composting are higher than those before composting. Therefore, HGT cannot be ruled out. Moreover, there is a clear positive correlation between MGEs and ARGs, which further proves that the potential of HGT to spread and concentrate ARGs cannot be ignored. Abundant and enriched ARGs can still be detected after composting, which indicates that direct application of composted organic fertilizers in the field may lead to the spread of ARGs in soils. Organic fertilizers from different sources have different abundances and diversities of ARGs due to the influence of different livestock and feeding sources. Although composting can effectively reduce the abundance of ARGs, the changes in MGEs are limited, so HGT fails to prevent ARG proliferation and its impact cannot be underestimated.

## Supplementary Materials

The following are available online at https://www.mdpi.com/2076-2607/8/2/268/s1.
