# Supplementary material for: Effects of Composting Different Types of Organic Fertilizer on the Microbial Community Structure and Antibiotic Resistance Genes
Source: Microorganisms. 2020 Feb 17;8(2):268. doi: 10.3390/microorganisms8020268 (PMC7074733; doi:10.3390/microorganisms8020268)
Supplement: Supplementary file 1 [file microorganisms-08-00268-s001.pdf]

Supporting Information for

Effects of composting different types of organic fertilizer on the microbial community structure and antibiotic resistance genes

Figures:

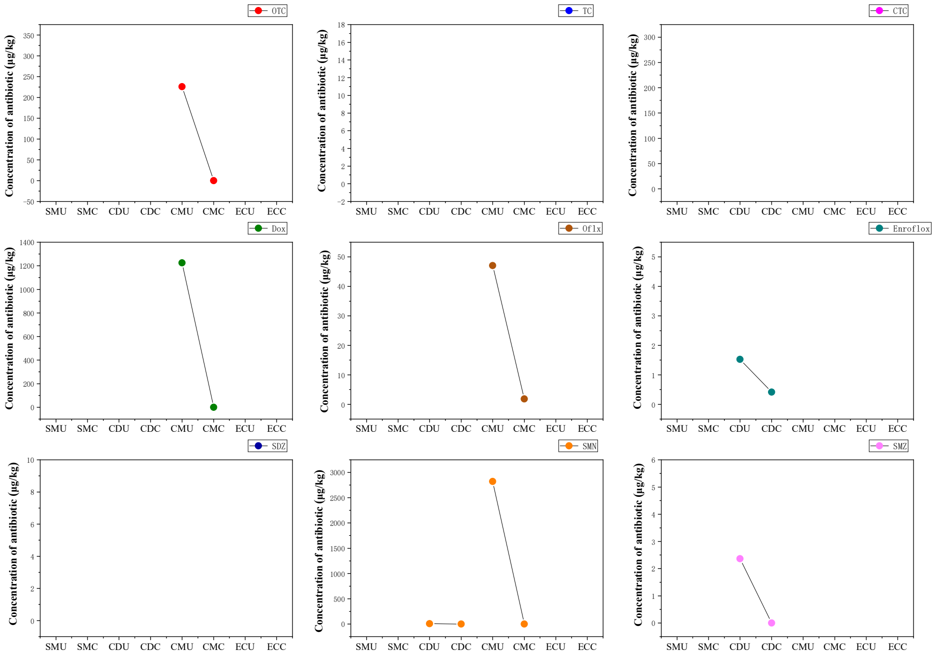

Figure S1. Changes in antibiotic residues before and after composting; unmarked items were not measured. (sulfadiazine (SDZ), oxytocin (OTC), sulfamethazine (SMZ), chlortetracycline (CTC), sulfamethoxypyridazine (SMN), TC, ofloxacin (Oflox), doxycycline (Dox) and enrofloxacin (Enroflox)).

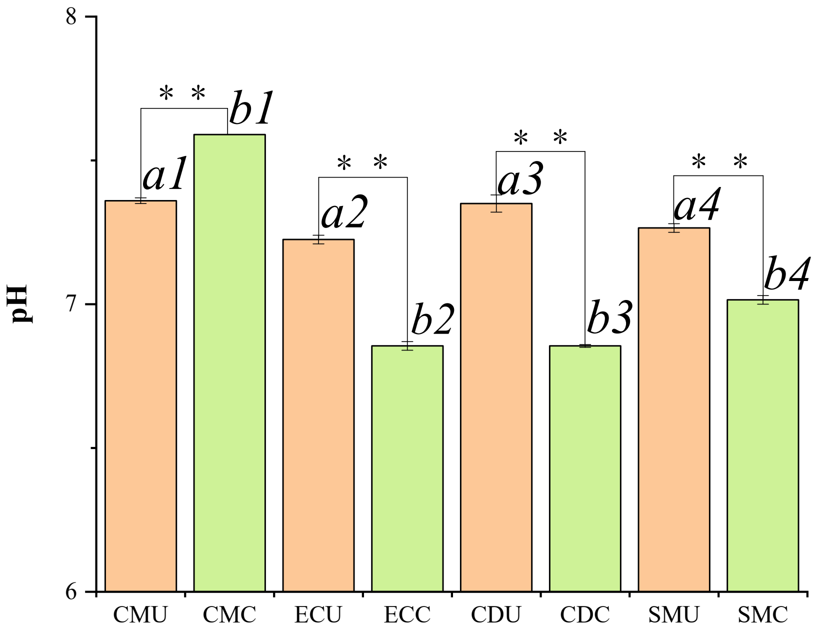

Figure S2. Change in pH before and after composting of organic fertilizers. Each value was a mean of three replicates. The same letter + different numbers within a group indicate significant differences at p <0.001 (ANOVA test; n = 3). ** (p < 0.01) on the bar indicates a statistically significant difference.

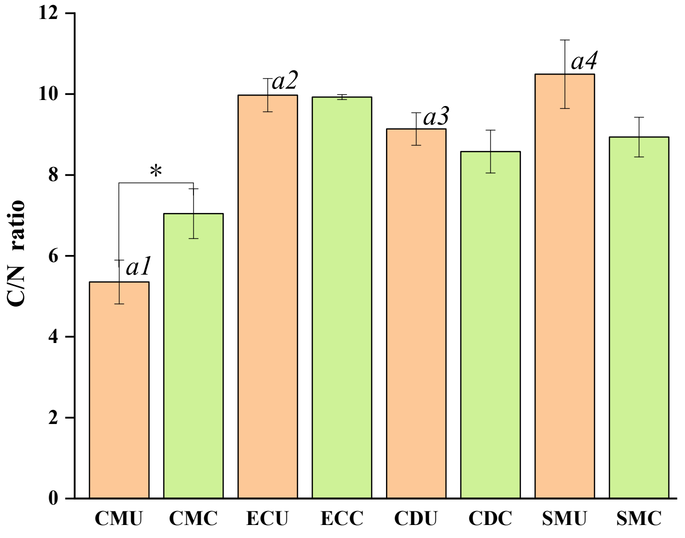

Figure S3. Differences in the C/N ratio before and after composting of five organic

fertilizers. The same letter + different numbers within a group indicate significant differences at p <0.001 (ANOVA test; n = 3). * (p < 0.05) on the bar indicates a statistically significant difference.

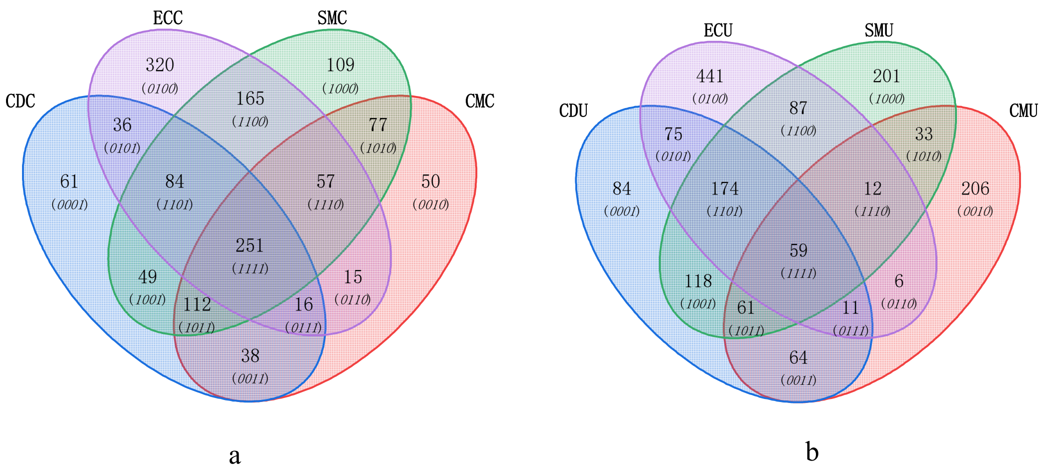

Figure S4. (a) Venn diagram showing the common and unique OTUs in organic fertilizer after composting. (b) Venn diagram showing the common and unique OTUs in uncomposted organic fertilizers. The binary-like numbers in the brackets in the figure indicate the inclusion relationship of different groups, 1 means include, 0 means not, and position indicates the group where it is located.

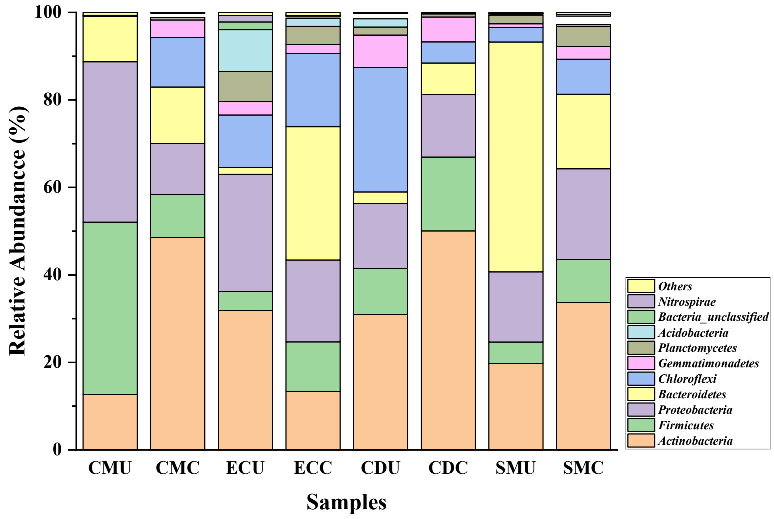

Figure S5. The effect of composting on the composition of organic fertilizer flora. Composition of the flora at the phylum level (mean, n = 3). Only the most abundant taxa (> 1% genus) are displayed (mean, n = 3).

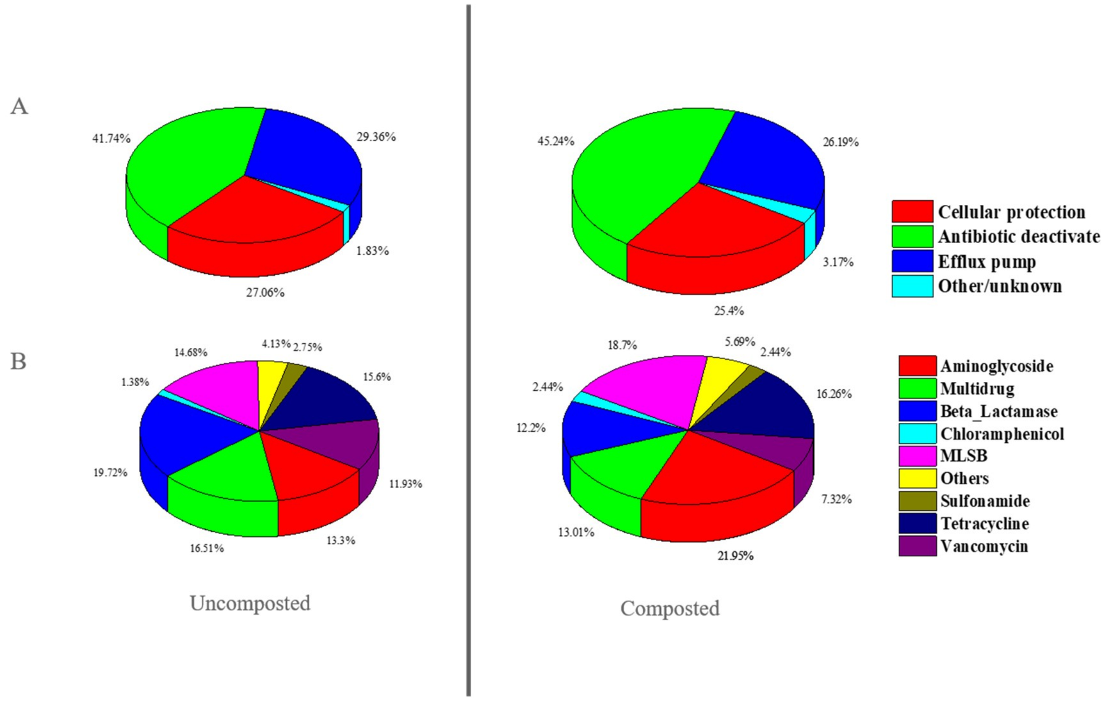

Figure S6. Resistance genes detected in all samples were classified based on (A) the mechanism of resistance and (B) the antibiotic to which they confer resistance. MLSB (Macrolide−Lincosamide−Streptogramin B).

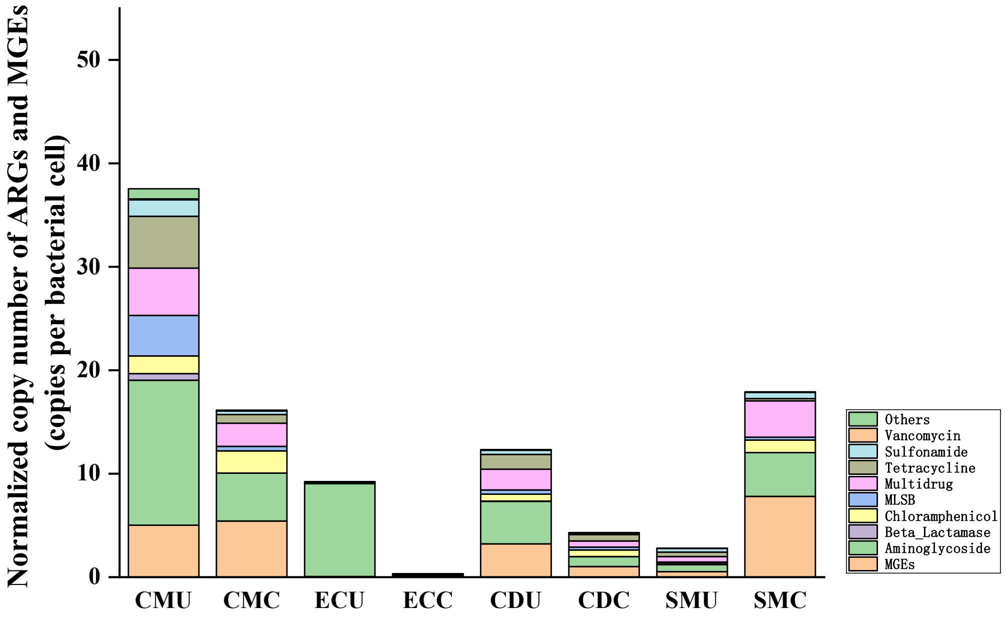

Figure S7. Normalized abundance of ARGs and MGE. Data shown in the figure are from three replicates and represented as the mean.

Table S1. Analysis of physicochemical properties.

Samples	C/N ratio	pH	NO_3^--N	NH_4^+-N
CMU	5.35±0.54	7.36	13.77	53.71
CMC	7.04±0.62	7.59	13.99	53.20
ECU	9.97±0.41	6.92	14.07	6.07
ECC	9.92±0.06	6.79	14.04	4.14
CDU	9.14±0.40	7.35	13.81	41.10
CDC	8.58±0.53	6.85	14.17	54.30
SMU	10.49±0.85	7.26	14.11	9.36
SMC	8.94±0.49	7.01	14.15	4.97

Table S2. The tested 296 target genes in this study (MLSB = Macrolide-Lincosamide-Streptogramin B resistance).

#Gene Name	Forward Primer	Reverse Primer	Classification of Antibiotic Resistance	The Mechanism of Resistance
16S rRNA	GGGTTGCGCTCGTTGC	ATGGYTGTCGTCAGCTCGTG		
aac	CCCTGCGTTGTGGCTATGT	TTGGCCACGCCAATCC	Aminoglycoside	antibiotic deactivate
aac(6')I1	GACCGGATTAAGGCCGATG	CTTGCCTTGATATTCAGTTTTTATAACCA	Aminoglycoside	antibiotic deactivate
aac(6')-Ib(aka aacA4)-01	GTTTGAGAGGCAAGGTACCGTAA	GAATGCCTGGCGTGTTTGA	Aminoglycoside	antibiotic deactivate
aac(6')-Ib(aka aacA4)-02	CGTCGCCGAGCAACTTG	CGGTACCTTGCCTCTCAAACC	Aminoglycoside	antibiotic deactivate
aac(6')-Ib(aka aacA4)-03	AGAAGCACGCCCGACACTT	GCTCTCCATTCAGCATTGCA	Aminoglycoside	antibiotic deactivate
aac(6')-II	CGACCCGACTCCGAACAA	GCACGAATCCTGCCTTCTCA	Aminoglycoside	antibiotic deactivate
aac(6')-Iy	GCTTTGCGGATGCCTCAAT	GGAGAACAAAAATACCTTCAAGGAAA	Aminoglycoside	antibiotic deactivate
aacA/aphD	AGAGCCTTGGGAAGATGAAGTTT	TTGATCCATACCATAGACTATCTCATCA	Aminoglycoside	antibiotic deactivate
aacC	CGTCACTTATTCGATGCCCTTAC	GTCGGGCGCGGCATA	Aminoglycoside	antibiotic deactivate
aacC1	GGTCGTGAGTTCGGAGACGTA	GCAAGTTCCCGAGGTAATCG	Aminoglycoside	antibiotic deactivate
aacC2	ACGGCATTCTCGATTGCTTT	CCGAGCTTCACGTAAGCATTT	Aminoglycoside	antibiotic deactivate
aacC4	CGGCGTGGGACACGAT	AGGGAACCTTTGCCATCAACT	Aminoglycoside	antibiotic deactivate
aadA-01	GTTGTGCACGACGACATCATT	GGCTCGAAGATACCTGCAAGAA	Aminoglycoside	antibiotic deactivate
aadA-02	CGAGATTCTCCGCGCTGTA	GCTGCCATTCTCCAAATTGC	Aminoglycoside	antibiotic deactivate
aadA1	AGCTAAGCGCGAACTGCAAT	TGGCTCGAAGATACCTGCAA	Aminoglycoside	antibiotic deactivate
aadA-1-01	AAAAGCCCGAAGAGGAACTTG	CATCTTTCACAAAGATGTTGCTGTCT	Aminoglycoside	antibiotic deactivate
aadA-1-02	CGGAATTGAAAAAACTGATCGAA	ATACCGGCTGTCCGTCATTT	Aminoglycoside	antibiotic deactivate

aadA2-01	ACGGCTCCGCAGTGGAT	GGCCACAGTAACCAACAAATCA	Aminoglycoside	antibiotic deactivate
aadA2-02	CTTGTCGTGCATGACGACATC	TCGAAGATACCCGCAAGAATG	Aminoglycoside	antibiotic deactivate
aadA2-03	CAATGACATTCTTGCGGGTATC	GACCTACCAAGGCAACGCTATG	Aminoglycoside	antibiotic deactivate
aadA5-01	ATCACGATCTTGCGATTTTGCT	CTGCGGATGGGCCTAGAAG	Aminoglycoside	antibiotic deactivate
aadA5-02	GTTCTTGCTCTTGCTCGCATT	GATGCTCGGCAGGCAAAC	Aminoglycoside	antibiotic deactivate
aadA9-01	CGCGGCAAGCCTATCTTG	CAAATCAGCGACCGCAGACT	Aminoglycoside	antibiotic deactivate
aadA9-02	GGATGCACGCTTGGATGAA	CCTCTAGCGGCCGGAGTATT	Aminoglycoside	antibiotic deactivate
aadD	CCGACAACATTTCTACCATCCTT	ACCGAAGCGCTCGTCGTATA	Aminoglycoside	antibiotic deactivate
aadE	TACCTTATTGCCCTTGGAAGAGTTA	GGAACTATGTCCCTTTTAATTCTACAATCT	Aminoglycoside	antibiotic deactivate
acrA-01	CAACGATCGGACGGGTTTC	TGGCGATGCCACCGTACT	Multidrug	efflux pump
acrA-02	GGTCTATCACCCTACGCGCTATC	GCGCGCACGAACATACC	Multidrug	efflux pump
acrA-03	CAGACCCGCATCGCATATT	CGACAATTTCGCGCTCATG	Multidrug	efflux pump
acrA-04	TACTTTGCGCGCCATCTTC	CGTGCGCGAACGAACAT	Multidrug	efflux pump
acrA-05	CGTGCGCGAACGAACA	ACTTTGCGCGCCATCTTC	Multidrug	efflux pump
acrB-01	AGTCGGTGTTCGCCGTTAAC	CAAGGAAACGAACGCAATACC	Multidrug	efflux pump
acrF	GCGGCCAGGCACAAAA	TACGCTCTTCCCACGGTTTC	Multidrug	efflux pump
acrR-01	GCGCTGGAGACACGACAAC	GCCTTGCTGCGAGAACAAA	Multidrug	efflux pump
acrR-02	GATGATACCCCCTGCTGTGAGA	ACCAAACAAGAAGCGCAAGAA	Multidrug	efflux pump
adeA	CAGTTCGAGCGCCTATTTCTG	CGCCCTGACCGACCAAT	Multidrug	efflux pump
ampC/blaDHA	TGGCCGCAGCAGAAAGA	CCGTTTTATGCACCCAGGAA	Beta_Lactamase	antibiotic deactivate
ampC-01	TGGCGTATCGGGTCAATGT	CTCCACGGGCCAGTTGAG	Beta_Lactamase	antibiotic deactivate
ampC-02	GCAGCACGCCCCGTAA	TGTACCCATGATGCGCGTACT	Beta_Lactamase	antibiotic deactivate
ampC-04	TCCGGTGACGCGACAGA	CAGCACGCCGGTGAAAGT	Beta_Lactamase	antibiotic deactivate
ampC-05	CTGTTCGAGCTGGGTTCTATAAGTAAA	CAGTATCTGGTCACCGGATCGT	Beta_Lactamase	antibiotic deactivate
ampC-06	CCGCTCAAGCTGGACCATAC	CCATATCCTGCACGTTGGTTT	Beta_Lactamase	antibiotic deactivate

ampC-07	CCGCCCAGAGCAAGGACTA	GCTCGACTTCACGCCGTAAG	Beta_Lactamase	antibiotic deactivate
ampC-09	CAGCCGCTGATGAAAAAATATG	CAGCGAGCCCACTTCGA	Beta_Lactamase	antibiotic deactivate
aph	TTTCAGCAAGTGGATCATGTTAAAAT	CCAAGCTGTTTCCACTGTTTTTC	Aminoglycoside	antibiotic deactivate
aph(2')-Id-01	TGAGCAGTATCATAAGTTGAGTGAAAAG	GACAGAACAATCAATCTCTATGGAATG	Aminoglycoside	antibiotic deactivate
aph(2')-Id-02	TAAGGATATACCGACAGTTTTGGAAA	TTTAATCCCTCTTCATACCAATCCATA	Aminoglycoside	antibiotic deactivate
aph6ia	CCCATCCCATGTGTAAGGAAA	GCCACCGCTTCTGCTGTAC	Aminoglycoside	antibiotic deactivate
aphA1(aka kanR)	TGAACAAGTCTGGAAAGAAATGCA	CCTATTAATTTCCCCTCGTCAAAAA	Aminoglycoside	antibiotic deactivate
bacA-01	CGGCTTCGTGACCTCGTT	ACAATGCGATACCAGGCAAAT	Others	antibiotic deactivate
bacA-02	TTCCACGACACGATTAAGTCATTG	CGGCTCTTTCGGCTTCAG	Others	antibiotic deactivate
bla1	GCAAGTTGAAGCGAAAGAAAAGA	TACCAGTATCAATCGCATATACACCTAA	Beta_Lactamase	antibiotic deactivate
bla-ACC-1	CACACAGCTGATGGCTTATCTAAAA	AATAAACGCGATGGGTTCCA	Beta_Lactamase	antibiotic deactivate
blaCMY	CCGCGGCGAAATTAAGC	GCCACTGTTTGCCTGTCAGTT	Beta_Lactamase	antibiotic deactivate
blaCMY2-01	AAAGCCTCAT GGGTGCATAAA	ATAGCTTTTGTTTGCCAGCATCA	Beta_Lactamase	antibiotic deactivate
blaCMY2-02	GCGAGCAGCCTGAAGCA	CGGATGGGCTTGTCCTCTT	Beta_Lactamase	antibiotic deactivate
blaCTX-M-01	GGAGGCGTGACGGCTTTT	TTCAGTGCGATCCAGACGAA	Beta_Lactamase	antibiotic deactivate
blaCTX-M-02	GCCGCGGTGCTGAAGA	ATCGGATTATAGTTAACCAGGTCAGATTT	Beta_Lactamase	antibiotic deactivate
blaCTX-M-03	CGATACCACCACGCCGTTA	GCATTGCCCAACGTCAGATT	Beta_Lactamase	antibiotic deactivate
blaCTX-M-04	CTTGGCGTTGCGCTGAT	CGTTCATCGGCACGGTAGA	Beta_Lactamase	antibiotic deactivate
blaCTX-M-05	GCGATAACGTGGCGATGAAT	GTCGAGACGGAACGTTTCGT	Beta_Lactamase	antibiotic deactivate
blaCTX-M-06	CACAGTTGGTGACGTGGCTTAA	CTCCGCTGCCGGTTTTATC	Beta_Lactamase	antibiotic deactivate
blaGES	GCAATGTGCTCAACGTTCAAG	GTGCCTGAGTCAATTCTTTCAAAG	Beta_Lactamase	antibiotic deactivate

blaIMP-01	AACACGGTTTGGTGGTTCTTGTA	GCGCTCCACAAACCAATTG	Beta_Lactamase	antibiotic deactivate
blaIMP-02	AAGGCAGCATTTCCTCTCATTTT	GGATAGATCGAGAATTAAGCCACTCT	Beta_Lactamase	antibiotic deactivate
bla-L1	CACCGGGTTACCAGCTGAAG	GCGAAGCTGCGCTTGTAGTC	Beta_Lactamase	antibiotic deactivate
blaMOX/blaCMY	CTATGTCAATGTGCCGAAGCA	GGCTTGTCCTCTTTCGAATAGC	Beta_Lactamase	antibiotic deactivate
blaOCH	GGCGACTTGCGCCGTAT	TTTTCTGCTCGGCCATGAG	Beta_Lactamase	antibiotic deactivate
blaOKP	GCCGCCATCACCATGAG	GGTGACGTTGTCACCGATCTG	Beta_Lactamase	antibiotic deactivate
blaOXA1/blaOXA30	CGGATGGTTTGAAGGGTTTATTAT	TCTTGGCTTTTATGCTTGATGTTAA	Beta_Lactamase	antibiotic deactivate
blaOXA10-01	CGCAATTATCGGCCTAGAAACT	TTGGCTTTCCGTCCCATTT	Beta_Lactamase	antibiotic deactivate
blaOXA10-02	CGCAATTATCGGCCTAGAAACT	TTGGCTTTCCGTCCCATTT	Beta_Lactamase	antibiotic deactivate
blaOXY	CGTTCAGGCGGCAGGTT	GCCGCGATATAAGATTTGAGAATT	Beta_Lactamase	antibiotic deactivate
blaPAO	CGCCGTACAACCGGTGAT	GAAGTAATGCGGTTCTCCTTTCA	Beta_Lactamase	antibiotic deactivate
blaPER	TGCTGGTTGCTGTTTTTGTGA	CCTGCGCAATGATAGCTTCAT	Beta_Lactamase	antibiotic deactivate
blaPSE	TTGTGACCTATTCCCCTGTAATAGAA	TGCGAAGCACGCATCATC	Beta_Lactamase	antibiotic deactivate
blaROB	GCAAAGGCATGACGATTGC	CGCGCTGTTGTCGCTAAA	Beta_Lactamase	antibiotic deactivate
blaSFO	CCGCCGCCATCCAGTA	GGGCCGCCAAGATGCT	Beta_Lactamase	antibiotic deactivate
blaSHV-01	TCCCATGATGAGCACCTTTAAA	TTCGTCACCGGCATCCA	Beta_Lactamase	antibiotic deactivate
blaSHV-02	CTTTCCCATGATGAGCACCTTT	TCCTGCTGGCGATAGTGGAT	Beta_Lactamase	antibiotic deactivate
blaTEM	AGCATCTTACGGATGGCATGA	TCCTCCGATCGTTGTCAGAAGT	Beta_Lactamase	antibiotic deactivate
blaTLA	ACACTTTGCCATTGCTGTTTATGT	TGCAAATTTCGGCAATAATCTTT	Beta_Lactamase	antibiotic deactivate
blaVEB	CCCGATGCAAAGCGTTATG	GAAAGATTCCCTTTATCTATCTCAGACAA	Beta_Lactamase	antibiotic deactivate
blaVIM	GCACTTCTCGCGGAGATTG	CGACGGTGATGCGTACGTT	Beta_Lactamase	antibiotic deactivate
blaZ	GGAGATAAAGTAACAAATCCAGTTAGATATGA	TGCTTAATTTTCCATTTGCGATAAG	Beta_Lactamase	antibiotic deactivate

carB	GGAGTGAGGCTGACCGTAGAAG	ATCGGCGAAACGCACAAA	MLSB	efflux pump
catA1	GGGTGAGTTTCACCAGTTTTGATT	CACCTTGTCGCCTTGCGTATA	Others	antibiotic deactivate
catB3	GCACTCGATGCCTTCCAAAA	AGAGCCGATCCAAACGTCAT	Others	antibiotic deactivate
catB8	CACTCGACGCCTTCCAAAG	CCGAGCCTATCCAGACATCATT	Others	antibiotic deactivate
ceoA	ATCAACACGGACCAGGACAAG	GGAAAGTCCGCTCACGATGA	Multidrug	efflux pump
cepA	AGTTGCGCAGAACAGTCCTCTT	TCGTATCTTGCCCGTCGATAAT	Beta_Lactamase	antibiotic deactivate
cfiA	GCAGCGTTGCTGGACACA	GTTCGGGATAAACGTGGTGACT	Beta_Lactamase	antibiotic deactivate
cfr	GCAAAATTCAGAGCAAGTTACGAA	AAAATGACTCCCAACCTGCTTTAT	Others	antibiotic deactivate
cfxA	TCATTCCTCGTTCAAGTTTTCAGA	TGCAGCACCAAGAGGAGATGT	Beta_Lactamase	antibiotic deactivate
cIntI-1(class1)	GGCATCCAAGCAGCAAG	AAGCAGACTTGACCTGA	Integron	integrase
cmeA	GCAGCAAAGAAGAAGCACCAA	AGCAGGGTAAGTAAAACTAAGTGGTAAATCT	Multidrug	efflux pump
cmlA1-01	TAGGAAGCATCGGAACGTTGAT	CAGACCGAGCACGACTGTTG	Chloramphenicol	efflux pump
cmlA1-02	AGGAAGCATCGGAACGTTGA	ACAGACCGAGCACGACTGTTG	Chloramphenicol	efflux pump
cmr	CGGCATCGTCAGTGGAATT	CGGTTCCGAAAAAGATGGAA	Multidrug	efflux pump
cmx(A)	GCGATCGCCATCCTCTGT	TCGACACGGAGCCTTGGT	Chloramphenicol	efflux pump
cphA-01	GCGAGCTGCACAAGCTGAT	CGGCCCAGTCGCTCTTC	Beta_Lactamase	antibiotic deactivate
cphA-02	GTGCTGATGGCGAGTTTCTG	GGTGTGGTAGTTGGTGTTGATCAC	Beta_Lactamase	antibiotic deactivate
dfrA1	GGAATGGCCCTGATATTCCA	AGTCTTGCGTCCAACCAACAG	Sulfonamide	antibiotic deactivate
dfrA12	CCTCTACCGAACCGTCACACA	GCGACAGCGTTGAAACAACTAC	Sulfonamide	antibiotic deactivate
emrD	CTCAGCAGTATGGTGGTAAGCATT	ACCAGGCGCCGAAGAAC	Multidrug	efflux pump
ereA	CCTGTGGTACGGAGAATTCATGT	ACCGCATTCGCTTTGCTT	MLSB	antibiotic deactivate
ereB	GCTTTATTTCAGGAGGCGGAAT	TTTTAAATGCCACAGCACAGAATC	Others	antibiotic deactivate

erm(34)	GCGCGTTGACGACGATTT	TGGTCATACTCGACGGCTAGAAC	MLSB	cellular protection
erm(35)	TTGAAAACGATGTTGCATTAAGTCA	TCTATAATCACAACTAACCACTTGAACGT	MLSB	cellular protection
erm(36)	GGCGGACCGACTTGCAT	TCTGCGTTGACGACGGTTAC	MLSB	cellular protection
ermA	TTGAGAAGGGATTTGCGAAAAG	ATATCCATCTCCACCATTAATAGTAAACC	MLSB	cellular protection
ermA/ermTR	ACATTTTACCAAGGAACTTGTGGAA	GTGGCATGACATAAACCTTCATCA	MLSB	cellular protection
ermB	TAAAGGGCATTTAACGACGAAACT	TTTATACCTCTGTTTGTTAGGGAATTGAA	MLSB	cellular protection
ermC	TTTGAAATCGGCTCAGGAAAA	ATGGTCTATTTCAATGGCAGTTACG	MLSB	cellular protection
ermF	CAGCTTTGGTTGAACATTTACGAA	AAATTCCTAAAATCACAACCGACAA	MLSB	cellular protection
ermJ/ermD	GGACTCGGCAATGGTCAGAA	CCCCGAAACGCAATATAATGTT	MLSB	cellular protection
ermK-01	GTTTGATATTGGCATTGTCAGAGAAA	ACCATTGCCGAGTCCACTTT	MLSB	cellular protection
ermK-02	GAGCCGCAAGCCCCTTT	GTGTTTCATTTGACGCGGAGTAA	MLSB	cellular protection
ermT-01	GTTCACTAGCACTATTTTTAATGACAGAAGT	GAAGGGTGTCTTTTTAATACAATTAACGA	MLSB	cellular protection
ermT-02	GTAAAATCCCTAGAGAATACTTTCATCCA	TGAGTGATATTTTTGAAGGGTGTCTT	MLSB	cellular protection
ermX	GCTCAGTGGTCCCCATGGT	ATCCCCCCGTCAACGTTT	MLSB	cellular protection
ermY	TTGTCTTTGAAAGTGAAGCAACAGT	TAACGCTAGAGAACGATTTGTATTGAG	MLSB	cellular protection
fabK	TTTCAGCTCAGCACTTTGGTCAT	AAGGCATCTTTTTCAGCCAGTTC	Others	antibiotic deactivate
floR	ATTGTCTTCACGGTGTCCGTTA	CCGCGATGTCGTCGAACT	Multidrug	efflux pump
folA	CGAGCAGTTCCTGCCAAAG	CCCAGTCATCCGGTTCATAATC	Sulfonamide	antibiotic deactivate
fosB	TCACTGTAACTAATGAAGCATTAGACCAT	CCATCTGGATCTGTAAAGTAAAGAGATC	Others	antibiotic deactivate
fosX	GATTAAGCCATATCACTTTAATTGTGAAAG	TCTCCTTCCATAATGCAAATCCA	Others	antibiotic deactivate

fox5	GGTTTGCCGCTGCAGTTC	GCGGCCAGGTGACCAA	Beta_Lactamase	antibiotic deactivate
imiR	CCGGACTAGAGCTTCATGTAAGC	CCCACGCGGTACTCTTGTAAA	Others	other/unknown
intI-1(clinic)	CGAACGAGTGGCGGAGGGTG	TACCCGAGAGCTTGGCACCCA	Integron	integrase
IS613	AGGTTCGGACTCAATGCAACA	TTCAGCACATACCGCCTTGAT	Transposase	transposase
lmrA-01	TCGACGTGACCGTAGTGAACA	CGTGACTACCCAGGTGAGTTGA	MLSB	efflux pump
lnuA-01	TGACGCTCAACACACTCAAAAA	TTCATGCTTAAGTTCCATACGTGAA	MLSB	antibiotic deactivate
lnuB-01	TGAACATAATCCCCTCGTTTAAAGAT	TAATTGCCCTGTTTCATCGTAAATAA	MLSB	antibiotic deactivate
lnuB-02	AAAGGAGAAGGTGACCAATACTCTGA	GGAGCTACGTCAAACAACCAGTT	MLSB	antibiotic deactivate
lnuC	TGGTCAATATAACAGATGTAAACCAGATTT	CACCCCAGCCACCATCAA	MLSB	antibiotic deactivate
marR-01	GCGGCGTACTGGTGAAGCTA	TGCCCTGGTCGTTGATGA	Multidrug	efflux pump
matA/mel	TAGTAGGCAAGCTCGGTGTTGA	CCTGTGCTATTTTAAGCCTTGTTTCT	MLSB	efflux pump
mdetl1	ATACAGCAGTGGATATTGGTTTAATTGT	TGCATAAGGTGAATGTTCCATGA	Multidrug	efflux pump
mdtA	CCTAACGGGCGTGACTTCA	TTCACCTGTTTCAAGGGTCAAA	MLSB	efflux pump
mdtE/yhiU	CGTCGGCGCACTCGTT	TCCAGACGTTGTACGGTAACCA	Multidrug	efflux pump
mecA	GGTTACGGACAAGGTGAAATACTGAT	TGTCTTTTAATAAGTGAGGTGCGTTAATA	Beta_Lactamase	cellular protection
mefA	CCGTAGCATTGGAACAGCTTTT	AAACGGAGTATAAGAGTGCTGCAA	MLSB	efflux pump
mepA	ATCGGTCGCTCTTCGTTCAC	ATAAATAGGATCGAGCTGCTGGAT	Multidrug	efflux pump
mexA	AGGACAACGCTATGCAACGAA	CCGGAAAGGGCCGAAAT	Multidrug	efflux pump
mexD	TTGCCACTGGCTTTCATGAG	CACTGCGGAGAACTGTCTGTAGA	Multidrug	efflux pump
mexE	GGTCAGCACCGACAAGGTCTAC	AGCTCGACGTACTTGAGGAACAC	Multidrug	efflux pump
mexF	CCGCGAGAAGGCCAAGA	TTGAGTTCGGCGGTGATGA	Multidrug	efflux pump

mphA-01	CTGACGCGCTCCGTGTT	GGTGGTGCATGGCGATCT	MLSB	antibiotic deactivate
mphA-02	TGATGACCCTGCCATCGA	TTCGCGAGCCCCTCTTC	MLSB	antibiotic deactivate
mphB	CGCAGCGCTTGATCTTGTAG	TTACTGCATCCATACGCTGCTT	MLSB	antibiotic deactivate
mphC	CGTTTGAAGTACCGAATTGGAAA	GCTGCGGGTTTGCCTGTA	MLSB	antibiotic deactivate
msrA-01	CTGCTAACACAAGTACGATTCCAAAT	TCAAGTAAAGTTGTCTTACCTACACCATT	MLSB	efflux pump
msrC-01	TCAGACCGGATCGGTTGTC	CCTATTTTTTGGAGTCTTCTCTCTAATGTT	MLSB	efflux pump
mtrC-01	GGACGGGAAGATGGTCCAA	CGTAGCGTTCCGGTTCGAT	Multidrug	efflux pump
mtrC-02	CGGAGTCCATCGACCATTTG	ATCGTCGGCAAGGAGAATCA	Multidrug	efflux pump
mtrD-02	GGTCGGCACGCTCTTGTC	TGAAGAATTTGCGCACCACTAC	Multidrug	efflux pump
mtrD-03	CCGCCAAGCCGATATAGACA	GGCCGGGTTGCCAAA	Multidrug	efflux pump
ndm-1	ATTAGCCGCTGCATTGAT	CATGTCGAGATAGGAAGTG	Beta_Lactamase	antibiotic deactivate
nimE	TGCGCCAAGATAGGGCATA	GTCGTGAATTCGGCAGGTTTA	Others	other/unknown
nisB	GGGAGAGTTGCCGATGTTGTA	AGCCACTCGTTAAAGGGCAAT	Others	other/unknown
oleC	CCCGGAGTCGATGTTCGA	GCCGAAGACGTACACGAACAG	MLSB	efflux pump
oprD	ATGAAGTGGAGCGCCATTG	GGCCACGGCGAACTGA	Multidrug	efflux pump
oprJ	ACGAGAGTGGCGTCGACAA	AAGGCGATCTCGTTGAGGAA	Multidrug	efflux pump
pbp	CCGGTGCCATTGGTTTAGA	AAAATAGCCGCCCCAAGATT	Beta_Lactamase	cellular protection
pbp2x	TTTCATAAGTATCTGGACATGGAAGAA	CCAAAGGAAACTTGCTTGAGATTAG	Beta_Lactamase	cellular protection
Pbp5	GGCGAACTTCTAATTAATCCTATCCA	CGCCGATGACATTCTTCTTATCTT	Beta_Lactamase	cellular protection
penA	AGACGGTAACGTATAACTTTTTGAAAGA	GCGTGTAGCCGGCAATG	Beta_Lactamase	cellular protection
pikR1	TCGACATGCGTGACGAGATT	CCGCGAATTAGGCCAGAA	MLSB	cellular protection
pikR2	TCGTGGGCCAGGTGAAGA	TTCCCCTTGCCGGTGAA	MLSB	cellular protection
pmrA	TTTGCAGGTTTTGTTCCTAATGC	GCAGAGCCTGATTTCTCCTTTG	Multidrug	efflux pump

pncA	GCAATCGAGGCGGTGTTC	TTGCCGCAGCCAATTCA	Others	other/unknown
putitive multidrug	AATTTTGCCGATTATTGCTGAAA	GATTGTCATCATTCGTTTATCACCAA	Multidrug	efflux pump
qac	CAATAATAACCGAAATAATAGGGACAAGTT	AATAAGTGTTCCTAGTGTTGGCCATAG	Multidrug	efflux pump
qacA	TGGCAATAGGAGCTATGGTGTTT	AAGGTAACACTATTTTCGGTCCAAATC	Multidrug	efflux pump
qacA/qacB	TTTAGGCAGCCTCGCTTCA	CCGAATCCAAATAAAACCCAATAA	Multidrug	efflux pump
qacEdelta1-01	TCGCAACATCCGCATTAAAA	ATGGATTTCAGAACCAGAGAAAGAAA	Multidrug	efflux pump
qacEdelta1-02	CCCCTTCCGCCGTTGT	CGACCAGACTGCATAAGCAACA	Multidrug	efflux pump
qacH-01	GTGGCAGCTATCGCTTGGAT	CCAACGAACGCCCACAA	Multidrug	efflux pump
qacH-02	CATCGTGCTTGTGGCAGCTA	TGAACGCCCAGAAGTCTAGTTTT	Multidrug	efflux pump
qnrA	AGGATTTCTCACGCCAGGATT	CCGCTTTCAATGAAACTGCAA	Others	other/unknown
rarD-02	TGACGCATCGCGTGATCT	AAATTTTCTGTGGCGTCTGAATC	Multidrug	efflux pump
sat4	GAATGGGCAAAGCATAAAAACTTG	CCGATTTTGAAACCACAATTATGATA	Others	antibiotic deactivate
sdeB	CACTACCGCTTCCGCACTTAA	TGAAAAAACGGGAAAAGTCCAT	Multidrug	efflux pump
spcN-01	AAAAGTTCGATGAAACACGCCTAT	TCCAGTGGTAGTCCCCGAATC	Aminoglycoside	antibiotic deactivate
spcN-02	CAGAATCTTCCTGAAAAGTTTGATGAA	CGCAGACACGCCGAATC	Aminoglycoside	antibiotic deactivate
speA	GCAAGAGGTATTTGCTCAACAAGA	CAGGGTCACCCTCATAAAGAAAA	Others	other/unknown
str	AATGAGTTTTGGAGTGTCTCAACGTA	AATCAAAACCCCTATTAAAGCCAAT	Aminoglycoside	antibiotic deactivate
strA	CCGGTGGCATTTGAGAAAAA	GTGGCTCAACCTGCGAAAAG	Aminoglycoside	antibiotic deactivate
strB	GCTCGGTCGTGAGAACAATCT	CAATTTCGGTCGCCTGGTAGT	Aminoglycoside	antibiotic deactivate
sul1	CAGCGCTATGCGCTCAAG	ATCCCGCTGCGCTGAGT	Sulfonamide	cellular protection
sul2	TCATCTGCCAAACTCGTCGTTA	GTCAAAGAACGCCGCAATGT	Sulfonamide	cellular protection

sulA/folP-01	CAGGCTCGTAAATTGATAGCAGAAG	CTTTCCTTGCGAATCGCTTT	Sulfonamide	cellular protection
sulA/folP-03	CACGGCTTCGGCTCATGT	TGCCATCCTGTGACTAGCTACGT	Sulfonamide	cellular protection
tet(32)	CCATTACTTCGGACAACGGTAGA	CAATCTCTGTGAGGGCATTTAACA	Tetracycline	cellular protection
tet(34)	CTTAGCGCAAACAGCAATCAGT	CGGTGATACAGCGCGTAAACT	Tetracycline	other/unknown
tet(35)	ACCCCATGACGTACCTGTAGAGA	CAACCCACACTGGCTACCAGTT	Tetracycline	other/unknown
tet(36)-01	AGAATACTCAGCAGAGGTCAGTTCCT	TGGTAGGTCGATAACCCGAAAAT	Tetracycline	cellular protection
tet(36)-02	TGCAGGAAAGACCTCCATTACAG	CTTTGTCCACACTTCCACGTACTATG	Tetracycline	cellular protection
tet(37)	GAGAACGTTGAAAAGGTGGTGAA	AACCAAGCCTGGATCAGTCTCA	Tetracycline	other/unknown
tetA-01	GCTGTTTGTTCTGCCGGAAA	GGTTAAGTTCCTTGAACGCAAACT	Tetracycline	efflux pump
tetA-02	CTCACCAGCCTGACCTCGAT	CACGTTGTTATAGAAGCCGCATAG	Tetracycline	efflux pump
tetB-01	AGTGCGCTTTGGATGCTGTA	AGCCCCAGTAGCTCCTGTGA	Tetracycline	efflux pump
tetB-02	GCCCAGTGCTGTTGTTGTCAT	TGAAAGCAAACGGCCTAAATACA	Tetracycline	efflux pump
tetC-01	CATATCGCAATACATGCGAAAAA	AAAGCCGCGGTAAATAGCAA	Tetracycline	efflux pump
tetC-02	ACTGGTAAGGTAAACGCCATTGTC	ATGCATAAACCAGCCATTGAGTAAG	Tetracycline	efflux pump
tetD-01	TGCCGCGTTTGATTACACA	CACCAGTGATCCCGGAGATAA	Tetracycline	efflux pump
tetD-02	TGTCATCGCGCTGGTGATT	CATCCGCTTCCGGGAGAT	Tetracycline	efflux pump
tetE	TTGGCGCTGTATGCAATGAT	CGACGACCTATGCGATCTGA	Tetracycline	efflux pump
tetG-01	TCAACCATTGCCGATTCGA	TGGCCCGGCAATCATG	Tetracycline	efflux pump
tetG-02	CATCAGCGCCGGTCTTATG	CCCCATGTAGCCGAACCA	Tetracycline	efflux pump
tetH	TTTGGGTCATCTTACCAGCATTAA	TTGCGCATTATCATCGACAGA	Tetracycline	efflux pump
tetJ	GGGTGCCGCATTAGATTACCT	TCGTCCAATGTAGAGCATCCATA	Tetracycline	efflux pump

tetK	CAGCAGTCATTGGAAAATTATCTGATTATA	CCTTGTACTAACCTACCAAAAATCAAAATA	Tetracycline	efflux pump
tetL-01	AGCCCGATTTATTCAAGGAATTG	CAAATGCTTTCCCCCTGTTCT	Tetracycline	efflux pump
tetL-02	ATGGTTGTAGTTGCGCGCTATAT	ATCGCTGGACCGACTCCTT	Tetracycline	efflux pump
tetM-01	CATCATAGACACGCCAGGACATAT	CGCCATCTTTTGCAGAAATCA	Tetracycline	cellular protection
tetM-02	TAATATTGGAGTTTTAGCTCATGTTGATG	CCTCTCTGACGTTCTAAAAGCGTATTAT	Tetracycline	cellular protection
tetO-01	ATGTGGATACTACAACGCATGAGATT	TGCCTCCACATGATATTTTTCCT	Tetracycline	cellular protection
tetPA	AGTTGCAGATGTGTATAGTCGTAAACTATCTATT	TGCTACAAGTACGAAAACAAAACTAGAA	Tetracycline	efflux pump
tetPB-01	ACACCTGGACACGCTGATTTT	ACCGTCTAGAACGCGGAATG	Tetracycline	cellular protection
tetPB-02	TGATACACCTGGACACGCTGAT	CGTCCAAAACGCGGAATG	Tetracycline	cellular protection
tetPB-03	TGGGCGACAGTAGGCTTAGAA	TGACCCTACTGAAACATTAGAAATATACCT	Tetracycline	cellular protection
tetPB-04	AGTGGTGCAAATACTGAAAAAGTTGT	TTTGTTCCTTCGTTTTGGACAGA	Tetracycline	cellular protection
tetPB-05	CTGAAGTGGAGCGATCATTCC	CCCTCAACGGCAGAAATAACTAA	Tetracycline	cellular protection
tetQ	CGCCTCAGAAGTAAGTTCATACACTAAG	TCGTTCATGCGGATATTATCAGAAT	Tetracycline	cellular protection
tetR-02	CGCGATAGACGCCTTCGA	TCCTGACAACGAGCCTCCTT	Tetracycline	efflux pump
tetR-03	CGCGATGGAGCAAAAGTACAT	AGTGAAAAACCTTGTTGGCATAAAA	Tetracycline	efflux pump
tetS	TTAAGGACAAACTTTCTGACGACATC	TGTCTCCCATTGTTCTGGTTCA	Tetracycline	cellular protection
tetT	CCATATAGAGGTTCCACCAAATCC	TGACCCTATTGGTAGTGGTTCTATTG	Tetracycline	cellular protection
tetU-01	GTGGCAAAGCAACGGATTG	TGCGGGCTTGCAAAACTATC	Tetracycline	other/unknown
tetV	GCGGGAACGACGATGTATATC	CCGCTATCTCACGACCATGAT	Tetracycline	efflux pump

tetX	AAATTTGTTACCGACACGGAAGTT	CATAGCTGAAAAAATCCAGGACAGTT	Tetracycline	other/unknown
tnpA-01	CATCATCGGACGGACAGAATT	GTCGGAGATGTGGGTGTAGAAAGT	Transposase	transposase
tnpA-02	GGGCGGGTCGATTGAAA	GTGGGCGGGATCTGCTT	Transposase	transposase
tnpA-03	AATTGATGCGGACGGCTTAA	TCACCAAACTGTTTATGGAGTCGTT	Transposase	transposase
tnpA-04	CCGATCACGGAAAGCTCAAG	GGCTCGCATGACTTCGAATC	Transposase	transposase
tnpA-05	GCCGCACTGTCGATTTTTATC	GCGGGATCTGCCACTTCTT	Transposase	transposase
tnpA-07	GAAACCGATGCTACAATATCCAATTT	CAGCACCGTTTGCAGTGTAAG	Transposase	transposase
tolC-01	GGCCGAGAACCTGATGCA	AGACTTACGCAATTCCGGGTTA	Multidrug	efflux pump
tolC-02	CAGGCAGAGAACCTGATGCA	CGCAATTCCGGGTTGCT	Multidrug	efflux pump
tolC-03	GCCAGGCAGAGAACCTGATG	CGCAATTCCGGGTTGCT	Multidrug	efflux pump
Tp614	GGAAATCAACGGCATCCAGTT	CATCCATGCGCTTTTGTCTCT	Transposase	transposase
ttgA	ACGCCAATGCCAAACGATT	GTCACGGCGCAGCTTGA	Multidrug	efflux pump
ttgB	TCGCCCTGGATGTACACCTT	ACCATTGCCGACATCAACAAC	Multidrug	efflux pump
vanA	AAAAGGCTCTGAAAACGCAGTTAT	CGGCCGTTATCTTGTAAAAACAT	Vancomycin	cellular protection
vanB-01	TTGTCGGCGAAGTGGATCA	AGCCTTTTTCCGGCTCGTT	Vancomycin	cellular protection
vanB-02	CCGGTCGAGGAACGAAATC	TCCTCCTGCAAAAAAAGATCAAC	Vancomycin	cellular protection
vanC-01	ACAGGGATTGGCTATGAACCAT	TGACTGGCGATGATTTGACTATG	Vancomycin	cellular protection
vanC-03	AAATCAATACTATGCCGGGCTTT	CCGACCGCTGCCATCA	Vancomycin	cellular protection
vanC1	AGGCGATAGCGGGTATTGAA	CAATCGTCAATTGCTCATTTCC	Vancomycin	cellular protection
vanC2/vanC3	TTTGACTGTCGGTGCTTGTGA	TCAATCGTTTCAGGCAATGG	Vancomycin	cellular protection
vanG	ATTTGAATTGGCAGGTATACAGGTTA	TGATTTGTCTTTGTCCATACATAATGC	Vancomycin	cellular protection
vanHB	GAGGTTTCCGAGGCGACAA	CTCTCGGCGGCAGTCGTAT	Vancomycin	cellular protection
vanHD	GTGGCCGATTATACCGTCATG	CGCAGGTCATTCAGGCAAT	Vancomycin	cellular protection

vanRA-01	CCCTTACTCCCACCGAGTTTT	TTCGTCGCCCCATATCTCAT	Vancomycin	cellular protection
vanRA-02	CCACTCCGGCCTTGTCATT	GCTAACCACATTCCCCTTGTTTT	Vancomycin	cellular protection
vanRB	GCCCTGTCGGATGACGAA	TTACATAGTCGTCTGCCTCTGCAT	Vancomycin	cellular protection
vanRC	TGCGGGAAAAACTGAACGA	CCCCCCATACGGTTTTGATTA	Vancomycin	cellular protection
vanRC4	AGTGCTTTGGCTTATCTCGAAAA	TCCGGCAGCATCACATCTAA	Vancomycin	cellular protection
vanRD	TTATAATGGCAAGGATGCACTAAAGT	CGTCTACATCCGGAAGCATGA	Vancomycin	cellular protection
vanSA	CGCGTCATGCTTTCAAAATTC	TCCGCAGAAAGCTCAATTTGTT	Vancomycin	cellular protection
vanSB	GCGCGGCAAATGACAAC	TTTGCCATTTTATTCGCACTGT	Vancomycin	cellular protection
vanSC-02	GCCATCAGCGAGTCTGATGA	CAGCTGGGATCGTTTTTCCTT	Vancomycin	cellular protection
vanSE	TGGCCGAAGAAGCAGGAA	CAATAATACTCGTCAAAGGAGTTCTCA	Vancomycin	cellular protection
vanTC-01	CACACGCATTTTTTCCCATCTAG	CAGCCAACAGATCATCAAAACAA	Vancomycin	cellular protection
vanTC-02	ACAGTTGCCGCTGGTGAAG	CGTGGCTGGTCGATCAAAA	Vancomycin	cellular protection
vanTE	GTGGTGCCAAGGAAGTTGCT	CGTAGCCACCGCAAAAAAAT	Vancomycin	cellular protection
vanTG	CGTGTAGCCGTTCCGTTCTT	CGGCATTACAGGTATATCTGGAAA	Vancomycin	cellular protection
vanWB	CGGACAAAGATACCCCCTATAAAG	AAATAGTAAATTGCTCATCTGGCACAT	Vancomycin	cellular protection
vanWG	ACATTTTCATTTTGGCAGCTTGTAC	CCGCCATAAGAGCCTACAATCT	Vancomycin	cellular protection
vanXA	CGCTAAATATGCCACTTGGGATA	TCAAAAGCGATTCAGCCAACT	Vancomycin	cellular protection
vanXB	AGGCACAAAATCGAAGATGCTT	GGGTATGGCTCATCAATCAACTT	Vancomycin	cellular protection
vanXD	TAAACCGTGTTATGGGAACGAA	GCGATAGCCGTCCCATAAGA	Vancomycin	cellular protection
vanYB	GGCTAAAGCGGAAGCAGAAA	GATATCCACAGCAAGACCAAGCT	Vancomycin	cellular protection
vanYD-01	AAGGCGATACCCTGACTGTCA	ATTGCCGGACGGAAGCA	Vancomycin	cellular protection
vanYD-02	CAAACGGAAGAGAGGTCACTTACA	CGGACGGTAATAGGGACTGTTC	Vancomycin	cellular protection

vatB-01	GGAAAAAGCAACTCCATCTCTTGA	TCCTGGCATAACAGTAACATTCTGA	MLSB	antibiotic deactivate
vatB-02	TTGGGAAAAAGCAACTCCATCT	CAATCCACACATCATTTCCAACA	MLSB	antibiotic deactivate
vatC-01	CGGAAATTGGGAACGATGTT	GCAATAATAGCCCCGTTTCCTA	MLSB	antibiotic deactivate
vatC-02	CGATGTTTGGATTGGACGAGAT	GCTGCAATAATAGCCCCGTTT	MLSB	antibiotic deactivate
vatE-01	GGTGCCATTATCGGAGCAAAT	TTGGATTGCCACCGACAAT	MLSB	antibiotic deactivate
vatE-02	GACCGTCCTACCAGGCGTAA	TTGGATTGCCACCGACAATT	MLSB	antibiotic deactivate
vgaA-01	CGAGTATTGTGGAAAGCAGCTAGTT	CCCGTACCGTTAGAGCCGATA	MLSB	efflux pump
vgaA-02	GACGGGTATTGTGGAAAGCAA	TTTCCTGTACCATTAGATCCGATAATT	MLSB	efflux pump
vgb-01	AGGGAGGGTATCCATGCAGAT	ACCAAATGCGCCCGTTT	MLSB	efflux pump
vgbB-01	CAGCCGGATTCTGGTCCTT	TACGATCTCCATTCAATTGGGTAAA	MLSB	antibiotic deactivate
vgbB-02	ATACGAGCTGCCTAATAAAGGATCTT	TGTGAACCACAGGGCATTATCA	MLSB	antibiotic deactivate
yceE/mdtG-01	TGGCACAAAATATCTGGCAGTT	TTGTGTGGCGATAAGAGCATTAG	Multidrug	efflux pump
yceE/mdtG-02	TTATCTGTTTTCTGCTCACCTTCTTTT	GCGTGGTGACAAACAGGCTTA	Multidrug	efflux pump
yceL/mdtH-01	TCGGGATGGTGGGCAAT	CGATAACCGAGCCGATGTAGA	Multidrug	efflux pump
yceL/mdtH-02	CGCGTGAAACCTTAAGTGCTT	AGACGGCTAAACCCCATATAGCT	Multidrug	efflux pump
yceL/mdtH-03	CTGCCGTTAAATGGATGTATGC	ACTCCAGCGGGCGATAGG	Multidrug	efflux pump
yidY/mdtL-01	GCAGTTGCATATCGCCTTCTC	CTTCCCGGCAAACAGCAT	Multidrug	efflux pump
yidY/mdtL-02	TGCTGATCGGGATTCTGATTG	CAGGCGCGACGAACATAAT	Multidrug	efflux pump

Table S3. Log number of Normalized gene copy numbers (copies per cell) (-: not detected).

#Gene Name	CMU	CMC	ECU	ECC	CDU	CDC	SMU	SMC
aac(6')-Ib(aka aacA4)-01	-1.14402	-2.39622	-4.08822	-	-1.73622	-2.42522	-2.78422	-2.27922
aac(6')-Ib(aka aacA4)-02	-0.82002	-2.21522	-	-3.81222	-1.70822	-2.30822	-2.88522	-2.14122
aac(6')-Ib(aka aacA4)-03	-1.06002	-2.13922	-3.86922	-3.62022	-1.54922	-2.15022	-2.65622	-2.09822
aac(6')-II	-1.22202	-2.84022	-	-4.33422	-2.01422	-	-3.25722	-
aacA/aphD	-	-2.72522	-4.20222	-	-2.54522	-3.15222	-3.44922	-2.82222
aacC	-	-	-3.83922	-	-	-	-	-
aacC1	-5.15202	-	-	-	-4.15722	-	-	-
aacC2	-1.13802	-3.66472	-3.66822	-	-3.14022	-3.93222	-3.41622	-3.70722
aacC4	-1.81902	-3.58822	-	-	-2.89522	-4.06522	-4.21322	-
aadA-01	-0.65802	-1.40322	-3.44622	-2.85522	-1.09722	-2.03622	-1.77022	-1.12822
aadA-02	-0.49902	-1.11222	-3.22722	-2.54322	-0.89622	-1.85022	-1.56622	-0.94822
aadA1	-0.31602	-0.88122	-3.00522	-2.58822	-0.89822	-1.54322	-1.55022	-0.65822
aadA-1-01	-0.76902	-1.99322	-3.89022	-3.44322	-2.52322	-2.79922	-4.12422	-
aadA-1-02	-	-1.73122	0.25278	-	-2.02122	-2.24122	-3.60622	-1.65522
aadA2-01	-0.89502	-1.03722	-3.92622	-3.02622	-1.28222	-1.66622	-2.09222	-0.81072
aadA2-02	-1.02702	-1.12322	-3.54822	-	-1.33022	-1.74622	-2.09722	-
aadA2-03	-0.54402	-0.81822	-3.30522	-2.57022	-0.79622	-1.46522	-1.54522	-
aadA5-01	-1.01202	-1.71822	-4.02822	-3.27222	-1.71822	-2.36922	-2.43722	-1.77522
aadA5-02	-1.21902	-1.72522	-	-3.05022	-1.63222	-2.18922	-2.37022	-1.65122
aadA9-01	-1.64802	-1.16322	-	-3.33222	-2.05822	-2.43322	-3.13022	-0.99522
aadA9-02	-1.87002	-1.31122	-3.87222	-3.53322	-2.20422	-2.49622	-3.23222	-1.38622
aadD	-1.04502	-1.03022	-3.85122	-3.71922	-1.84122	-2.72122	-3.06722	-1.57022

aadE	-1.08102	-1.33322	-3.22422	-3.33522	-1.70022	-1.71922	-3.10822	-1.79122
aph(2')-Id-01	-3.55002	-	-	-	-3.82122	-4.20622	-4.42922	-
aph(2')-Id-02	-2.37402	-3.78522	-	-	-3.36822	-4.09622	-	-3.58222
aphA1(aka kanR)	-0.88602	-2.82222	-	-4.04022	-2.14322	-3.02722	-	-2.21472
str	-	-3.20622	-	-4.69122	-3.43622	-3.81022	-3.77422	-3.91022
strA	-2.35302	-3.38822	-	-4.63122	-3.33522	-	-4.20822	-3.99672
strB	-0.84702	-1.98322	-3.19722	-3.29022	-1.47222	-2.75022	-2.69822	-2.03622
ampC/blaDHA	-2.78202	-	-	-	-3.73272	-	-	-4.58922
ampC-01	-4.25802	-	-	-	-	-	-	-4.52322
ampC-02	-2.26902	-	-	-	-	-	-4.28372	-
ampC-04	-4.97202	-	-3.94122	-	-	-4.06822	-4.05722	-
ampC-05	-4.40202	-	-	-	-4.08072	-	-	-
ampC-06	-4.77402	-	-	-	-4.08822	-	-	-
ampC-07	-5.66502	-	-	-	-	-	-	-
bla1	-	-	-4.03722	-3.34422	-	-	-	-
blaCMY	-3.30702	-	-	-	-	-	-	-
blaCMY2-01	-3.64002	-	-	-	-	-	-	-
blaCMY2-02	-5.11902	-	-	-	-	-	-	-4.77922
blaCTX-M-01	-2.78802	-	-	-	-	-	-	-
blaCTX-M-02	-2.55702	-	-4.53222	-	-	-	-	-
blaCTX-M-04	-	-	-3.31722	-	-4.22622	-	-4.21322	-
blaCTX-M-05	-2.78802	-	-	-	-	-	-	-
blaCTX-M-06	-4.57902	-	-	-	-	-	-	-
blaGES	-	-	-	-	-	-	-	-
blaMOX/blaCMY	-4.30302	-	-	-	-	-	-	-
blaOXA1/blaOXA30	-1.86402	-	-	-	-3.55522	-	-	-

blaOXA10-01	-1.65702	-	-4.11822	-	-3.44722	-4.04422	-4.19322	-
blaOXA10-02	-1.67502	-	-4.01622	-	-3.46422	-4.28722	-3.94322	-
blaOXY	-4.37802	-	-3.77622	-	-3.05022	-	-	-
blaPAO	-4.81602	-	-	-	-3.81322	-	-	-
blaPER	-2.26302	-	-	-	-	-	-	-
blaPSE	-1.64202	-	-4.38822	-	-3.20622	-4.15822	-4.07322	-
blaROB	-5.39502	-	-	-	-	-	-	-
blaSFO	-	-	-	-3.88422	-3.61222	-3.16522	-3.75272	-
blaSHV-01	-3.51402	-	-	-	-	-	-	-
blaSHV-02	-3.70902	-	-	-	-4.13022	-	-	-
blaTEM	-1.92402	-	-	-	-3.79722	-	-4.03422	-
blaVEB	-2.70702	-	-	-	-	-	-	-
blaVIM	-4.73502	-	-	-	-	-	-	-
blaZ	-3.92502	-	-	-	-	-	-	-4.76022
cepA	-4.68102	-	-	-	-	-	-	-
cfiA	-5.50302	-	-	-	-	-	-	-
cfxA	-2.52102	-	-	-4.88022	-4.33422	-	-	-4.76322
cphA-01	-	-	-3.45222	-	-3.19122	-	-3.96872	-
cphA-02	-	-	-	-	-	-4.01622	-	-
fox5	-	-	-2.82522	-	-3.01822	-3.33172	-3.09722	-
ndm-1	-	-	-	-	-	-	-	-4.66722
mecA	-4.21302	-	-	-	-	-	-	-
pbp	-2.93502	-	-	-	-	-	-	-
Pbp5	-1.92702	-	-	-	-4.05072	-	-	-4.27522
cmlA1-01	-2.17902	-2.35822	-4.15722	-4.20522	-2.31822	-3.23872	-2.02822	-2.16622
cmlA1-02	-2.13402	-2.32922	-	-4.16322	-2.21422	-3.05422	-1.91022	-2.09722

cmx(A)	-0.48702	-0.37622	-2.90922	-2.46522	-0.90522	-0.89722	-2.05622	-0.64222
ereA	-2.69202	-	-4.29222	-	-3.50522	-4.24222	-	-
lnuA-01	-	-	-4.59822	-	-4.06022	-	-3.53022	-3.87522
lnuB-01	-1.11702	-2.82222	-2.74722	-2.77422	-2.22622	-2.54222	-3.73822	-2.93222
lnuB-02	-1.51302	-3.40022	-3.11922	-3.23022	-2.68422	-2.94522	-4.19522	-3.36722
mphA-01	-	-	-	-	-	-3.83122	-3.94322	-
mphA-02	-	-	-	-	-2.79822	-	-	-
vatB-02	-	-	-	-	-	-	-	-
vatC-01	-5.57202	-	-4.08522	-4.90722	-4.14622	-	-	-4.95672
vatE-01	-1.37502	-3.56322	-3.88722	-4.77222	-3.77722	-3.70422	-4.45422	-4.52522
vatE-02	-1.75602	-3.77872	-	-	-3.77322	-3.91722	-	-
vgbB-01	-	-	-	-	-	-	-4.41722	-
vgbB-02	-5.49702	-	-	-	-	-4.29322	-	-
erm(34)	-	-	-	-	-3.76722	-	-	-
erm(35)	-2.29302	-3.48772	-	-	-3.16272	-	-4.49622	-3.05522
erm(36)	-2.41602	-2.62622	-4.19322	-	-2.37322	-3.66822	-4.02922	-
ermA	-1.76502	-2.06222	-	-	-2.73222	-3.13222	-	-
ermA/ermTR	-3.85902	-	-	-	-	-	-	-
ermB	-	-2.35922	-4.47822	-	-2.67822	-3.60422	-3.60422	-2.67522
ermC	-2.09502	-2.21922	-	-	-2.81222	-3.50422	-4.11322	-
ermF	-0.44502	-1.59722	-4.16322	-3.64122	-1.66322	-2.28122	-1.98722	-1.53672
ermJ/ermD	-5.40402	-	-	-	-3.56222	-4.28872	-4.28522	-
ermK-01	-	-	-	-	-3.60522	-	-	-
ermT-01	-1.25802	-2.75522	-	-4.41522	-2.24922	-3.27422	-3.19322	-2.82672
ermT-02	-1.15902	-1.92822	-4.25322	-4.22622	-1.95222	-2.64722	-3.21822	-
ermX	-1.26102	-1.85322	-3.36222	-3.13122	-1.90422	-1.86022	-3.30622	-1.87022

ermY	-3.98202	-3.50122	-	-	-3.71022	-4.16022	-	-3.41622
matA/mel	-1.57602	-2.66922	-2.97522	-3.29922	-2.75522	-2.74522	-3.83722	-
mdtA	-4.05402	-	-	-	-	-	-	-
mefA	-1.74402	-2.16022	-2.59422	-2.75622	-2.37022	-2.15722	-2.71122	-2.18022
oleC	-3.72102	-2.85022	-2.30622	-	-2.95922	-1.73522	-2.63422	-
vgaA-01	-4.89102	-3.69922	-	-	-	-	-	-
vgb-01	-5.60802	-	-3.46722	-4.19922	-2.76522	-3.07522	-3.23322	-4.63122
acrA-01	-2.50902	-	-	-	-	-	-	-
acrA-02	-2.42202	-	-	-	-	-	-	-
acrA-04	-5.63502	-3.79222	-3.37122	-	-3.56022	-	-3.69722	-4.07622
acrA-05	-	-3.58522	-	-3.55422	-2.82222	-3.11722	-3.16522	-3.58422
acrB-01	-2.13402	-	-	-	-	-	-4.24922	-
acrF	-2.12802	-	-	-	-	-	-4.35722	-
acrR-01	-5.33502	-	-	-	-	-	-	-
acrR-02	-2.26302	-	-	-	-	-	-4.03922	-
adeA	-5.46702	-	-	-	-3.57722	-	-	-
ceoA	-	-	-3.63222	-	-3.80922	-2.79922	-	-
cmr	-2.00202	-3.48322	-	-	-3.98622	-4.27672	-	-
emrD	-	-	-4.19322	-	-	-	-	-
floR	-0.55002	-1.86622	-3.41922	-3.31722	-1.38322	-1.84622	-2.17222	-1.89522
marR-01	-	-	-4.46022	-	-	-	-	-
mdtE/yhiU	-2.21802	-	-4.60422	-	-	-	-4.44122	-
mepA	-	-	-	-	-3.78522	-4.11022	-4.30022	-
mexA	-	-	-	-	-3.90822	-	-	-
mexE	-	-	-	-	-3.98322	-	-	-
mexF	-	-1.49722	-	-	-1.23322	-1.62822	-1.48022	-

oprD	-4.88502	-	-3.03222	-	-2.85522	-3.62422	-3.62372	-
oprJ	-4.90002	-	-2.12922	-	-2.97622	-2.88322	-3.28472	-
putitive multidrug	-	-	-4.35222	-3.51222	-	-	-	-
qacEdelta1-01	-0.80202	-0.83822	-3.23322	-2.58522	-0.97422	-1.58822	-1.64322	-0.54522
qacEdelta1-02	-0.41502	-0.59222	-2.86122	-2.34822	-0.73322	-1.29122	-1.38622	-0.39372
qacH-01	-2.02002	-3.24522	-	-	-2.66772	-	-	-
qacH-02	-2.56902	-	-	-4.81122	-3.88622	-	-	-
rarD-02	-	-	-	-	-	-	-	-
tolC-01	-2.16102	-	-	-	-4.30572	-	-4.46072	-
tolC-02	-3.87402	-	-3.87522	-	-4.32222	-	-	-
tolC-03	-3.82602	-	-4.09422	-	-	-	-	-
ttgB	-5.52402	-	-4.32822	-	-3.23522	-	-	-
yceE/mdtG-01	-2.05902	-	-	-	-4.24272	-	-3.97322	-
yceL/mdtH-01	-	-	-3.98022	-	-	-	-	-
yceL/mdtH-02	-2.13102	-3.80122	-	-	-	-	-3.97322	-4.39122
yceL/mdtH-03	-2.37402	-	-	-	-	-	-4.26822	-
yidY/mdtL-02	-2.23902	-	-	-	-	-	-4.37372	-
bacA-02	-2.29602	-	-	-	-4.01622	-	-	-
catA1	-3.67602	-	-	-	-	-	-	-
catB3	-1.41702	-2.79822	-	-4.30122	-2.39822	-3.32422	-3.00622	-2.78522
catB8	-1.96602	-2.93022	-4.36122	-4.61622	-3.35622	-2.81422	-3.72322	-3.24922
cfr	-1.22202	-2.18222	-4.22022	-4.42722	-2.34422	-3.02822	-3.52322	-2.97722
ereB	-2.93202	-	-3.92622	-	-	-4.21222	-	-
sat4	-1.09902	-2.26222	-4.23222	-3.96222	-2.63522	-2.79422	-	-2.18922
pncA	-	-	-	-	-3.99072	-3.78222	-3.84122	-
qnrA	-3.64902	-	-	-	-	-	-	-4.58622

dfrA1	-0.99102	-2.39522	-3.56622	-3.73422	-1.75322	-2.70222	-2.59322	-2.16522
dfrA12	-1.84002	-3.59722	-	-	-3.34422	-	-	-
folA	-5.66802	-	-	-	-	-	-	-
sul2	-0.68802	-1.19722	-2.92422	-2.21322	-1.20222	-1.51322	-1.15722	-0.95022
sulA/folP-01	-	-	-4.32822	-	-	-	-	-
sulA/folP-03	-	-	-4.09122	-	-	-	-	-
tet(32)	-2.59302	-	-3.89322	-4.07622	-3.65022	-3.66422	-4.28622	-4.58322
tet(36)-01	-1.99002	-	-	-	-3.85722	-	-	-
tet(36)-02	-2.48202	-	-	-	-3.76022	-	-	-
tetM-01	-0.61302	-2.42022	-2.65422	-2.84622	-2.06222	-2.45522	-3.28922	-2.92722
tetM-02	-1.19202	-3.37522	-3.32922	-3.48522	-2.98922	-3.50922	-4.31622	-3.85522
tetO-01	-2.11602	-	-3.47322	-3.78822	-3.38822	-3.15922	-3.63222	-4.55122
tetPB-01	-	-	-3.67422	-3.62922	-	-	-	-
tetPB-02	-	-	-	-3.95922	-	-	-	-4.65822
tetPB-03	-2.46402	-	-3.14922	-3.33222	-2.13022	-2.75722	-3.84022	-
tetPB-05	-	-	-3.79422	-3.36222	-	-	-	-
tetQ	-1.65102	-	-	-	-3.55522	-	-	-
tetS	-2.01102	-	-	-	-3.99422	-	-	-
tetT	-1.49202	-2.67922	-3.58422	-3.30222	-2.87422	-2.98822	-3.39022	-2.93022
tetA-02	-1.30602	-2.52322	-3.57822	-3.47022	-1.64722	-3.27422	-2.82422	-
tetB-01	-1.57302	-	-	-	-3.75622	-	-	-
tetB-02	-1.42002	-3.55222	-4.11522	-4.90122	-3.43022	-3.99722	-4.01422	-3.90222
tetC-01	-1.97202	-	-	-	-	-	-	-
tetC-02	-1.81902	-	-	-	-	-	-	-
tetD-01	-3.56802	-	-	-	-	-	-	-
tetD-02	-3.73602	-	-	-	-	-	-	-

tetE	-4.23402	-	-	-	-	-	-	-
tetG-01	-1.39002	-1.48722	-3.43122	-2.59122	-1.25822	-1.75022	-1.70122	-
tetG-02	-1.59102	-1.40422	-	-2.46222	-0.98222	-1.42822	-1.47922	-
tetH	-	-	-	-	-	-	-	-
tetJ	-3.77802	-	-	-	-	-	-	-
tetK	-4.21602	-	-	-	-	-	-	-
tetL-01	-	-	-4.56822	-	-3.85722	-	-4.18172	-
tetL-02	-1.08402	-1.15622	-3.22722	-3.38922	-1.36922	-1.78372	-2.54022	-1.72722
tetPA	-3.25002	-	-3.71022	-4.17822	-2.57622	-3.32122	-	-
tetR-02	-1.71702	-2.68322	-	-	-1.97322	-3.50522	-3.11922	-2.36622
tetR-03	-1.58502	-	-	-	-3.78622	-	-4.50722	-4.42722
tetV	-	-	-3.93222	-	-	-3.65422	-3.22522	-
tet(34)	-	-	-	-	-	-4.17022	-	-
tetX	-0.57402	-1.83622	-3.95022	-3.65322	-1.54222	-1.37022	-1.58522	-1.70022
IS613	-1.19802	-3.06222	-	-	-3.27122	-	-4.50322	-3.00822
tnpA-01	-2.69202	-3.76822	-	-	-	-3.75022	-	-
tnpA-02	-1.10502	-2.31622	-	-	-1.70472	-2.92822	-2.47822	-
tnpA-03	-1.88802	-3.70622	-	-	-4.12522	-	-	-3.72822
tnpA-04	-0.46002	0.01978	-2.42622	-1.82922	-0.26622	-0.72122	-1.12022	0.16778
tnpA-05	-0.32502	-2.51822	-3.90822	-3.34722	-1.70222	-2.71022	-2.44522	-2.32222
tnpA-07	-1.85202	-3.09022	-	-4.23522	-2.98422	-3.26222	-	-3.29522
Tp614	-2.31702	-	-	-	-4.34322	-	-4.02272	-4.69722
cIntI-1(class1)	-2.87802	-3.25672	-	-3.89322	-2.72022	-2.85722	-2.74322	-2.83872
intI-1(clinic)	-2.18802	-1.53422	-2.11422	-	-1.21922	-2.05822	-1.68622	-1.08522
vanA	-5.20602	-	-3.58122	-	-4.06122	-4.10272	-	-
vanB-01	-4.92402	-	-	-	-	-4.04872	-	-

vanB-02	-5.66202	-	-	-	-	-	-	-
vanC-01	-2.80002	-	-	-	-4.29822	-	-	-
vanC-03	-2.63802	-	-	-4.04322	-3.24522	-	-3.42622	-3.72822
vanC1	-3.41202	-	-	-	-	-	-	-
vanC2/vanC3	-2.63202	-	-	-	-	-	-	-4.88172
vanHB	-	-	-4.12722	-4.90422	-	-	-	-
vanRA-01	-5.65902	-3.52422	-3.35622	-	-4.06422	-3.50922	-3.66622	-
vanRA-02	-5.34102	-	-4.10322	-4.78122	-	-4.20022	-	-
vanRB	-5.30502	-	-	-	-	-	-	-
vanRC	-	-	-	-	-4.10022	-	-4.35872	-
vanRC4	-3.23802	-	-	-	-	-	-4.53122	-
vanSA	-4.69302	-	-	-	-	-	-	-
vanSB	-4.91202	-	-	-	-	-	-	-
vanSC-02	-2.52102	-	-	-	-	-	-	-
vanTC-01	-2.95002	-	-	-	-	-	-	-
vanTC-02	-2.59302	-	-4.47822	-	-4.27122	-	-	-
vanTE	-3.09102	-	-	-	-4.11822	-4.18522	-	-
vanTG	-5.50302	-	-	-	-	-	-	-
vanWB	-5.56902	-	-	-	-	-	-	-
vanWG	-5.12502	-	-	-	-	-	-4.36622	-
vanXA	-5.17302	-	-	-	-	-	-	-
vanXB	-4.78302	-	-	-	-	-	-	-
vanXD	-	-3.12022	-3.17022	-	-3.32322	-3.26422	-3.86622	-
vanYB	-5.32302	-	-	-	-	-	-	-
